# The Interaction of Calcium-Sensing Receptor with KIF11 Enhances Cisplatin Resistance in Lung Adenocarcinoma via BRCA1/cyclin B1 pathway

**DOI:** 10.7150/ijbs.92046

**Published:** 2024-07-15

**Authors:** Fuhao Wang, Xing Fu, Ming Chang, Tianzi Wei, Risheng Lin, Haibo Tong, Xiao Zhang, Runzhu Yuan, Zhiqing Zhou, Xin Huang, Wei Zhang, Wenmei Su, Yi Lu, Zhen Liang, Jian Zhang

**Affiliations:** 1 School of Medicine, Southern University of Science and Technology, Shenzhen, Guangdong 518055, China.; 2 The First Affiliated Hospital, School of Medicine, Southern University of Science and Technology, Shenzhen 518055, China.; 3 Faculty of Health Sciences, University of Macau, Taipa, Macao SAR, 999078, China.; 4 Joint Laboratory of Guangdong-Hong Kong Universities for Vascular Homeostasis and Diseases, School of Medicine, Southern University of Science and Technology, Shenzhen, Guangdong 518055, China.; 5 School of Public Health and Emergency Management, Southern University of Science and Technology, Shenzhen 518055, Guangdong, China.; 6 Department of Pulmonary Oncology, Affiliated Hospital of Guangdong Medical University, Zhanjiang, China.

**Keywords:** lung adenocarcinoma, chemoresistance, CaSR, BRCA1, cyclin B1, KIF11.

## Abstract

Cisplatin (DDP) is commonly used in the treatment of non-small cell lung cancer (NSCLC), including lung adenocarcinoma (LUAD), and the primary cause for its clinical inefficacy is chemoresistance. Here, we aimed to investigate a novel mechanism of chemoresistance in LUAD cells, focusing on the calcium-sensing receptor (CaSR). In this study, high CaSR expression was detected in DDP-resistant LUAD cells, and elevated CaSR expression is strongly correlated with poor prognosis in LUAD patients receiving chemotherapy. LUAD cells with high CaSR expression exhibited decreased sensitivity to cisplatin, and the growth of DDP-resistant LUAD cells was inhibited by cisplatin treatment in combination with CaSR suppression, accompanied by changes in BRCA1 and cyclin B1 protein expression both *in vitro* and *in vivo*. Additionally, an interaction between CaSR and KIF11 was identified. Importantly, suppressing KIF11 resulted in decreased protein levels of BRCA1 and cyclin B1, enhancing the sensitivity of DDP-resistant LUAD cells to cisplatin with no obvious decrease in CaSR. Here, our findings established the critical role of CaSR in promoting cisplatin resistance in LUAD cells by modulating cyclin B1 and BRCA1 and identified KIF11 as a mediator, highlighting the potential therapeutic value of targeting CaSR to overcome chemoresistance in LUAD.

## Introduction

Lung cancer remains the deadliest form of cancer globally, with the highest number of fatalities in recent years regardless of gender 1. It has also been the second leading cause of cancer incidence globally in 2023 2, 3. Non-small cell lung cancer (NSCLC) represents the most widespread type of lung cancer, accounting for approximately 85% of cases 4. Research in the field of lung cancer treatment is of considerable importance and significance 5, 6. Chemotherapy has been crucial strategy 7, both in early 8 and in advanced 9, 10 NSCLC patients. Platinum chemotherapy is commonly used in the management of lung cancer and is presently acknowledged as the primary treatment option for lung cancer by the American Society of Clinical Oncology (ASCO) and the National Comprehensive Cancer Network (NCCN) 11, 12; it can be utilized as part of a combination drug strategy to lengthen the overall survival of patients and still prevails as the primary approach for the treatment of lung cancer. Cisplatin, which is widely acknowledged as the most extensively used platinum chemotherapy drug, is commonly employed for treating NSCLC 13, it hinders the DNA replication and repair processes of tumor cells, consequently restraining tumor growth and metastasis 14. However, the emergence of drug resistance is the primary cause of failure in current clinical treatments for NSCLC. Reversing drug resistance and improving the efficacy of cisplatin are ongoing challenges in clinical applications. Although previous studies have explored the mechanisms of cisplatin resistance in NSCLC, few definitive molecular markers have been identified to predict or reverse resistance 15. In the clinical management of lung cancer, foreknowledge of how the disease responds to cisplatin is crucial in selecting more efficacious treatments, allowing for significant strides in treating the condition.

Calcium-sensing receptor (CaSR), as a calcium sensor, regulates cell division and many fundamental biological processes 16. The effect of CaSR on tumors has been investigated in various human malignancies 17. Recent research indicates that higher CaSR expression in breast cancer may be associated with a worse prognosis and treatment outcome, independent of subtype 18, 19. In lung cancer, CaSR is also highly expressed in LUAD tissues, and the expression level thereof is associated with the degrees of cancer differentiation and metastasis 20. CaSR has also been reported as a potential biomarker for predicting bone metastasis and prognosis in lung cancer patients 21. Therapeutic employment of CaSR targeting can be considered in patients with increased CaSR expression in cancer cells, which exhibit increased tumor malignancy 22. Moreover, some reviews have reported that CaSR is associated with drug resistance 23, 24. Nevertheless, little is known about the role of CaSR in lung cancer and its contribution to chemoresistance in lung cancer. CaSR controls cell proliferation by modifying calcium channel expression 25, a key point in cell cycle 26. Cancer cells rely heavily on the G2/M phase for DNA repair 27 and the capacity for DNA damage repair is intimately connected with the cisplatin resistance observed in NSCLC 28. However, it remains unclear how CaSR affects the cell cycle and DNA damage repair in tumor cells. Therefore, further investigation is required to determine the significance of CaSR in cancer treatment.

Adenocarcinoma is the most prevalent histological subtype of NSCLC, accounting for a high proportion of NSCLC patients in China 29. Considering that the mechanism of cisplatin resistance varies among different types of tumors, we established two DDP-resistant LUAD cell sublines by progressively escalating the concentration of cisplatin. We observed high expression of CaSR in both DDP-resistant cell lines, indicating a potential association between CaSR and cisplatin resistance. Moreover, our data revealed a significant correlation between CaSR expression and prognosis in LUAD patients undergoing chemotherapy. Subsequently, we conducted a systematic investigation to elucidate the role of CaSR in the development of cisplatin resistance. Our findings offer new strategies for accurately predicting tumor chemosensitivity, reversing multidrug resistance, and gaining insights into the molecular mechanisms underlying chemoresistance in LUAD cells.

## Methods and materials

### Cell culture

A549 and H1299 cell lines were obtained from ATCC (Manassas, VA, USA). A549-DDP and H1299-DDP cell lines resistant to cisplatin were established by exposing the cells to progressive concentrations of cisplatin for approximately 2 months. Cells were cultured in RPMI 1640 (Gibco; Carlsbad, CA, USA) with 10% fetal bovine serum (FBS, Gibco) and 1% penicillin/streptomycin (Gibco) in an incubator with 5% CO_2_ at 37°C. The culture medium of DDP-resistant cells was supplemented with an additional 2 µM cisplatin. All cell lines were tested against contamination by mycoplasma.

### Drugs and inhibitors

Cisplatin (HY-17394) and NPS-2143 (HY-10007) were purchased from MedChem Express (Monmouth Junction, NJ, USA). A kinesin family member 11 (KIF11) inhibitor (EG5 Inhibitor V, trans-24; T11155) was purchased from TargetMol (Boston, MA, USA).

### Clinical samples

Tissue sections from patient clinical samples were purchased from Zhongke Guanghua intelligent biological technology co. (Xi'an, China).

### RNA-seq and data analysis

Total RNA was extracted using 1 mL TRIzol per 10 cm^2^ plate volume for each RNA sequencing (RNA-seq) sample. All RNA-seq samples were quality controlled and customized for analysis by Gene Denovo Biotechnology Co. (Guangzhou, China). DEseq2 software was utilized to analyze the distinctively expressed genes. Genes with false discovery rates (FDR) < 0.05 and |Log2(fold change)| > 1 were considered differentially expressed genes (DEGs), which were utilized for the subsequent enrichment analysis.

### Survival analysis

Survival and prognosis analysis was evaluated by the Kaplan-Meier plotter server 30, which contained independent datasets from the caBIG, GEO, and TCGA repositories.

### CCK8 assay

A total of 2000 cells in 100 µl of medium were seeded into 96-well plates. Then, 10 µl of Cell Counting Kit-8 (CCK8, TargetMol) reagent was added to the cells and incubated at 37°C for 2 h. The absorbance was measured at 450 nm using a microplate reader (BioTek; Vermont, USA). For cisplatin resistance detection, cisplatin concentrations were set to 0, 1, 2, 4, 8, 16, 32, or 64 µM.

### Clonogenicity assay

Cells were seeded into 6-well dishes at a concentration of 300 cells per dish. After two weeks of growth, the cells were treated with 4% cell fixative (Solarbio; Beijing, China) and stained with crystal violet (Solarbio) for colony counting.

### Western blotting

The total protein was lysed by sonication and the protocol used for western blotting was based on a previous report 31. The primary antibodies were listed in Table S1.

### Establishment of CaSR-overexpressing cell lines

To overexpress CaSR, the coding sequence of CaSR was subcloned. The protocol used for establishing the CaSR-overexpressing cell lines was based on a previous report 32.

### Cell cycle analysis

Cells from each sample were cultured for cell cycle analysis in 6-cm dishes. After rinsing with PBS, the cells were fixed by placing them in 75% pre-cooled ethanol overnight. After washing with PBS, staining was then performed at 37°C for 30 min (Beyotime; Shanghai, China) and was detected using an AFC2 acoustic focusing cytometer (Invitrogen; Carlsbad, CA, USA).

### Apoptosis analysis

A total of 2-5*10^6^ cells were collected for the purpose of apoptosis detection. The cells were digested with trypsin without EDTA and were prepared with the apoptosis detection kit (Yeasen; Shanghai, China). The samples were then detected using an AFC2 acoustic focusing cytometer (Invitrogen; Carlsbad, CA, USA).

### Glycolysis stress test

The Agilent Seahorse XF Glycolysis Stress Test Kit (Agilent; CA, USA) was employed to assess the principal parameters of glycolytic flux. The cells were examined on the Agilent Seahorse XF Pro Analyzer (Agilent; CA, USA).

### RNAi transfection

Lipofectamine RNAiMax reagent (Invitrogen, 13778150), Opti-MEM medium (Gibco, 31985-062), targeted siRNAs and non-targeted controls from General Biosystems Co. (Anhui, China) were transfected into cells. The working concentrations for all siRNAs were 10-20 nM, and the targeting sequences for each siRNA are listed in Table S2.

### Animal model

Female BALB/c nude mice at approximately 4 weeks of age were used for the tumorigenesis assay. Each mouse was subcutaneously injected with a total of 2 × 10^6^ A549-DDP cells on the right side of the back. Injections were performed when tumor diameters reached 4 mm, with 6 mice per group. The weights and tumor volumes of the mice were monitored, and injections were given twice a week for 1 month. Mice were euthanized at study termination, and tumors were removed and weighed. Sections of subcutaneous tumor were used for immunohistochemistry (IHC), and the remaining sections were stored in formalin. The animal protocol received approval from the Institutional Animal Ethics Committee at the Southern University of Science and Technology. All mice were maintained by facility technicians at the Laboratory Animal Center (SUSTech-JY2020140).

### Immunohistochemistry

For IHC detection, slides were incubated with the indicated primary antibodies (Table S1). Following incubation with the primary antibodies, the slides were subjected to a secondary antibody incubation using an IHC kit (Biotechnologies) for 20 min. The results were observed utilizing a digital pathology scanner (Leica Microsystems; Wetzlar, Germany). CaSR, KI67, BRCA1, and cyclin B1 positive cells were quantified by measuring staining intensity and percentage using ImageJ software.

### Molecular docking

The X-ray crystal structures of CaSR (7SIL) and KIF11 (1IIL) were retrieved from the Protein Data Bank. To ensure the accuracy of the docking results, the proteins were prepared using AutoDockTools-1.5.7^33^, ^34^.

### Immunoprecipitation and co-immunoprecipitation

A total of 1 × 10^7^ cells were lysed with NP-40 buffer (Beyotime) and centrifuged at 12000 rpm for 10 min. Antibodies (Table S1) were added, and the samples were shaken overnight at 4°C. Protein G Dynabeads (Invitrogen) were introduced and incubated for 4 h at 4°C, and the isolated samples were then collected for mass spectrometry and western blotting.

### Statistical analysis

The results of this study are presented as the means ± SEM. Statistically significant variations between two groups were evaluated utilizing an independent t-test, and comparisons across more than two groups were evaluated using one-way analysis of variance (ANOVA). Survival curves were developed from Kaplan-Meier estimates, and the log-rank test was used to perform survival analysis. *P* < 0.05 was considered statistically significant (* *P* < 0.05, ** *P* < 0.01, *** *P* < 0.001, **** *P* < 0.0001 and ns, not significant). R software and GraphPad Prism 9 were used for all statistical analyses and visualizations.

## Results

### CaSR was upregulated in DDP-resistant LUAD cells and associated with poor prognosis of chemotherapy in LUAD

**Figure 1:**
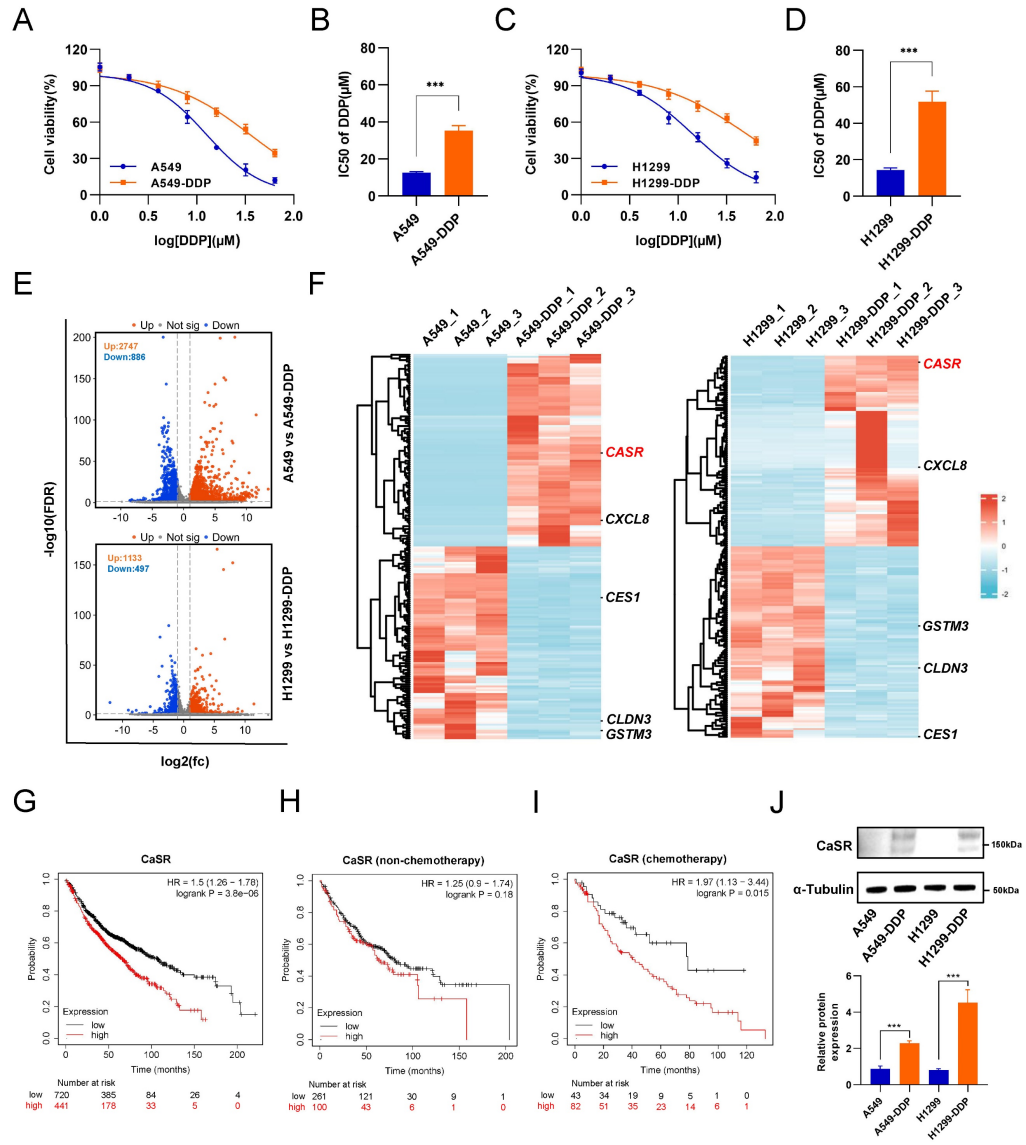
** CaSR was upregulated in DDP-resistant LUAD cells and associated with poor prognosis in LUAD.** Cell viability and the IC50 values of cisplatin in A549-DDP and parental A549 cell lines **(A and B)** were determined by CCK8 assay. Same with H1299-DDP and parental H1299 cell lines **(C and D)**. Volcano plot showed differentially expressed genes between parental and DDP-resistant LUAD cells **(E)**. Heat map showed the top 100 up-regulated and down-regulated differentially expressed genes (DEGs) from the two cisplatin-resistant cells and their parental cells **(F)**. Kaplan-Meier plots showed the correlation of CaSR with overall survival in LUAD **(G)**, including the prognosis receiving non-chemotherapy **(H)** and chemotherapy **(I)**. Western blotting results showed high CaSR expression in DDP-resistant LUAD cells **(J)**. Data are depicted as the mean ± SEM. * *P* < 0.05, ** *P* < 0.01, *** *P* < 0.001 and ns, not significant.

Cisplatin (DDP) remains the predominant therapeutic regimen in LUAD, overcoming cisplatin resistance will liberate its curative potential. Here, we established two LUAD cell lines, based on A549 and H1299 cell lines, with cisplatin resistance using progressively increasing concentrations of cisplatin (Fig. S1A). A comparison of the half-maximal inhibitory concentration (IC50) values revealed that the cisplatin resistance levels of the LUAD cell lines were significantly higher than those of the parental A549 and H1299 cells (Fig. 1A-D). To comprehensively investigate the molecular mechanism of LUAD resistance and identify potential targets to reverse resistance, we sequenced the transcriptomes of the parental and resistant A549 and H1299 cell lines. The results revealed 2747 upregulated genes and 886 downregulated genes in A549-DDP cells compared to parental cells and 1133 upregulated genes and 497 downregulated genes in H1299-DDP cells compared to parental cells (Fig. 1E). We conducted intersection analysis on the top 100 up-regulated and down-regulated differentially expressed genes (DEGs) from the two cisplatin-resistant cells and their parental cells, aiming to identify genes which were commonly up-regulated and down-regulated in A549-DDP and H1299-DDP cells. The results showed there were 3 co-upregulated genes (*Calcium-sensing receptor, CaSR; CXC motif chemokine ligand 8, CXCL8; and ARL14EPL*) and 6 co-downregulated genes (*glutathione S-transferase mu 3, GSTM3; CES4A; carboxylesterase 1, CES1; PPP1R3G; claudin 3, CLDN3; RGL3*) (Fig. S1B). Subsequently, we utilized the Kaplan-Meier plotter server to investigate the association between these genes and the prognosis of LUAD patients who received chemotherapy. Survival information was identified only for the *CXCL8, CaSR, CLDN3, GSTM3,* and *CES1* genes in LUAD patients who underwent chemotherapy intervention (Fig.1F, S1C). We observed that no statistically significant correlations were found between *CXCL8, CLDN3, GSTM3,* and LUAD chemotherapy prognosis (Fig. S1E-G). Patients with high expression of CES1 had a poor prognosis after chemotherapy (Fig. S1H), though high CES1 expression was not detected in our established DDP-resistant LUAD cells (Fig.1F). Therefore, we suppose that CES1 may have a role in other types of chemotherapy. We further focused on CaSR. Surprisingly, our analysis revealed a strong correlation between elevated CaSR expression and survival in LUAD patients (Fig. 1G). Moreover, we also found that high expression of CaSR was associated with a worse prognosis following chemotherapy in breast cancer patients who did not receive endocrine therapy (Fig. S2A) and in ovarian cancer patients who underwent suboptimal debulking surgery (Fig. S2B). Then we hypothesized that CaSR might be associated with chemotherapy prognosis. Upon further examination, we divided the data into two groups based on whether the patients underwent chemotherapy or not. Intriguingly, we found no significant variations in CaSR expression and prognosis among LUAD patients without a history of chemotherapy (Fig. 1H). However, among patients who received chemotherapy, those with high CaSR expression had a worse prognosis (Fig. 1I). According to the GEPIA database, there were some LUAD tumor tissues with high CaSR expression, although there was no statistical difference between tumor and normal tissues in LUAD at the overall level (Fig. S1D). Additionally, our study detected high levels of CaSR protein in our established DDP-resistant LUAD cells (Fig. 1J), and high CaSR expression was detected in clinical samples from LUAD patients with chemotherapy (Fig. S3), suggesting that CaSR may play a role in the progression of chemotherapy in LUAD patients.

### High expression of CaSR enhanced cisplatin resistance in LUAD cell lines

**Figure 2:**
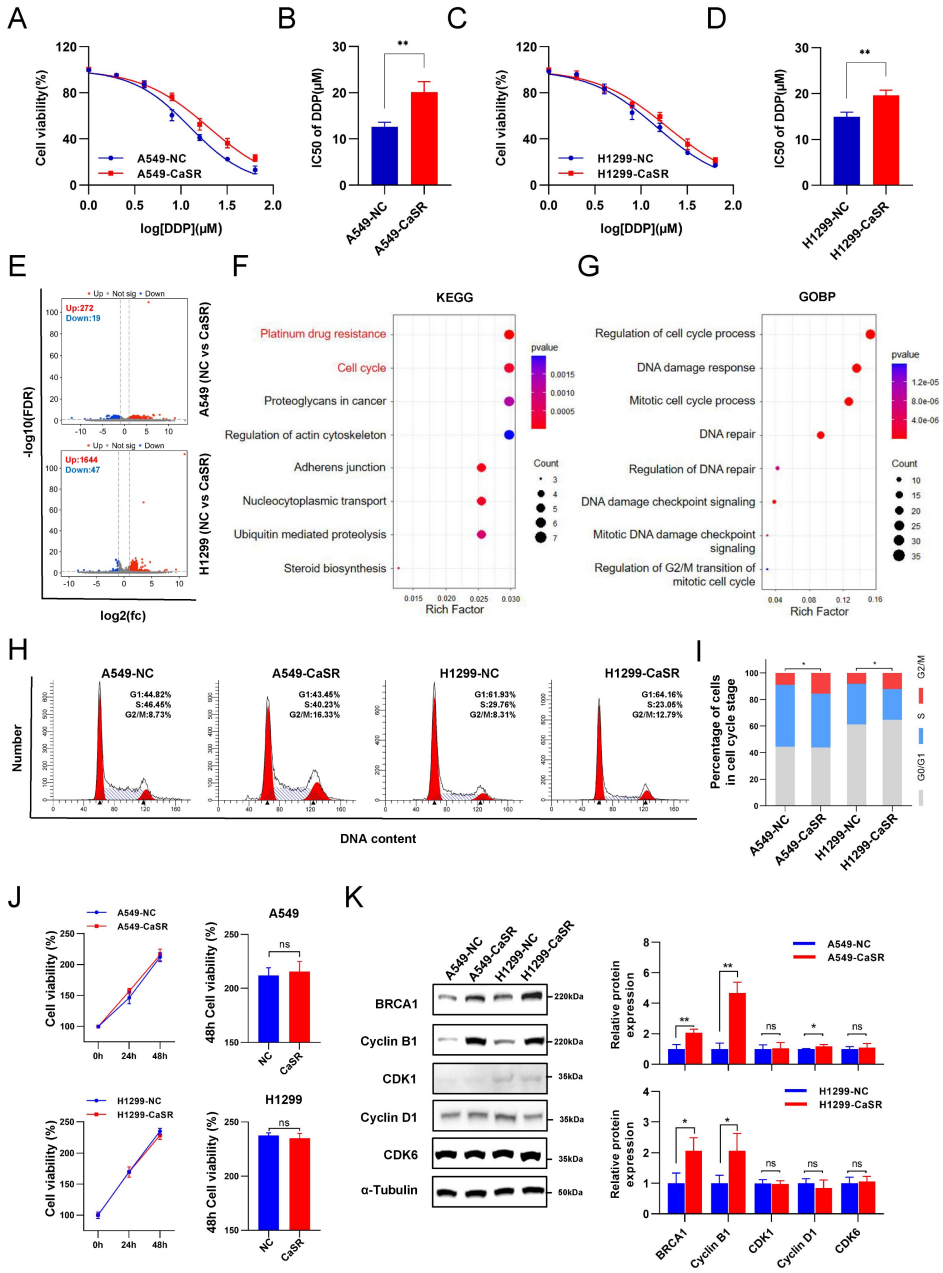
** Overexpression of CaSR affected the cell cycle and cisplatin resistance in LUAD cell lines.** Cell viability and the IC50 values of cisplatin in A549-NC and A549-CaSR cell lines **(A and B)**, H1299-NC and H1299-CaSR cell lines **(C and D)**. Volcano plot showed differentially expressed genes between CaSR-overexpressing cell lines and negative control cell lines **(E)**. KEGG enrichment analysis **(F)**, GO enrichment analysis **(G)**, and cell cycle analysis were presented **(H and I)**. CaSR overexpression did not affect the proliferation of LUAD cells, and the panels on the right showed the relative cell viability after 48 h **(J)**. BRCA1 and cell cycle related proteins were determined by western blotting between CASR-overexpressing cell lines and negative control cell lines **(K)**. Data are presented as the mean ± SEM. * *P* < 0.05, ** *P* < 0.01, *** *P* < 0.001, and ns, not significant.

As indicated by the data presented above, CaSR could potentially be linked to chemotherapy resistance in LUAD. To investigate the involvement of CaSR in the chemotherapeutic resistance of LUAD, we established CaSR-overexpressing A549 and H1299 cell lines using lentiviruses, with cells stably expressing empty vector used as a negative control (Fig. S4A). Indeed, we found that upregulation of CaSR expression increased cisplatin resistance in LUAD cells to a certain extent (Fig. 2A-D). We performed transcriptome sequencing on stable CaSR-overexpressing and control cell lines to further investigate the involvement of CaSR in the mechanism of chemoresistance. We found that A549 cells overexpressing CaSR had 272 upregulated genes compared to negative control cells. In H1299 cells overexpressing CaSR, 1644 genes were upregulated, and 47 genes were downregulated compared to negative control cells (Fig. 2E).

Moreover, we identified 235 co-upregulated genes and 1 co-downregulated gene (Fig. S4B). Enrichment analysis of the Kyoto Encyclopedia of Genes and Genomes (KEGG) signaling pathways revealed that CaSR-overexpressing LUAD showed significant changes in cisplatin drug resistance-related pathways compared to the negative control group (Fig. 2F), including BRCA1(Fig. S4C), which plays an important role in homology-directed repair and therapy resistance 35, and BRCA1 is also an important indicator for predicting the efficacy of chemotherapy in NSCLC 36. Meanwhile, the enrichment outcomes of Gene Ontology (GO), Reactome, and WikiPathways analyses highlighted the activation of numerous pathways linked to the cell cycle and DNA damage repair (Fig. 2G, S4D, E). These pathways are strongly associated with the development of cisplatin resistance. Based on the transcriptome sequencing results, significant changes in the cell cycle, especially in the G2/M phase, were observed in the CaSR-overexpressing cell lines compared to the NC group (Fig. 2H, I). CaSR overexpression did not have a significant impact on the proliferation of LUAD in either A549 or H1299 cell lines (Fig. 2J). Additionally, there was no significant change observed in the G1 phase (Fig. 2H). Therefore, we hypothesize that the increased percentage of cells in G2/M phase is not due to cell arrest, but rather a result of the cell's self-adaptation and self-regulation. To further investigate this, we examined the expression levels of CDK6 and cyclin D1, two important regulators of the G1 phases 37. However, we observed either inconsistent changes or no substantial alteration in their expression levels. Moreover, the G2/M phase-related proteins, cyclin-dependent kinase 1 (CDK1), cyclin B1 and breast cancer gene 1 (BRCA1) were also evaluated in the CaSR-overexpressing and control cell lines. We found a pronounced upregulation of BRCA1 in CaSR-overexpressing LUAD cells. Cyclin B1 expression was significantly increased in these cells, though there was no substantial change in CDK1 expression (Fig. 2K). As we know, cyclin B1/CDK1 plays a crucial role in regulating cell cycle progression. When the protein level of cyclin B1 reaches a certain level, it enters the nucleus and forms a complex with CDK1 to affect the cell cycle 38. Typically, cyclin B1 is the main regulated protein 39, and its increased presence may be closely related to the increase in the proportion of G2/M phase cells. Moreover, cyclin B1 stability is increased by interaction with BRCA1 40, which supports our results. As the majority of tumor cells depend on the G2/M checkpoint for DNA repair, the increase of BRCA1 and the elevated percentages of CaSR-overexpressing cells in the LUAD cell lines in G2/M phase imply that these cells possess a more adequate DNA repair process.

**Figure 3:**
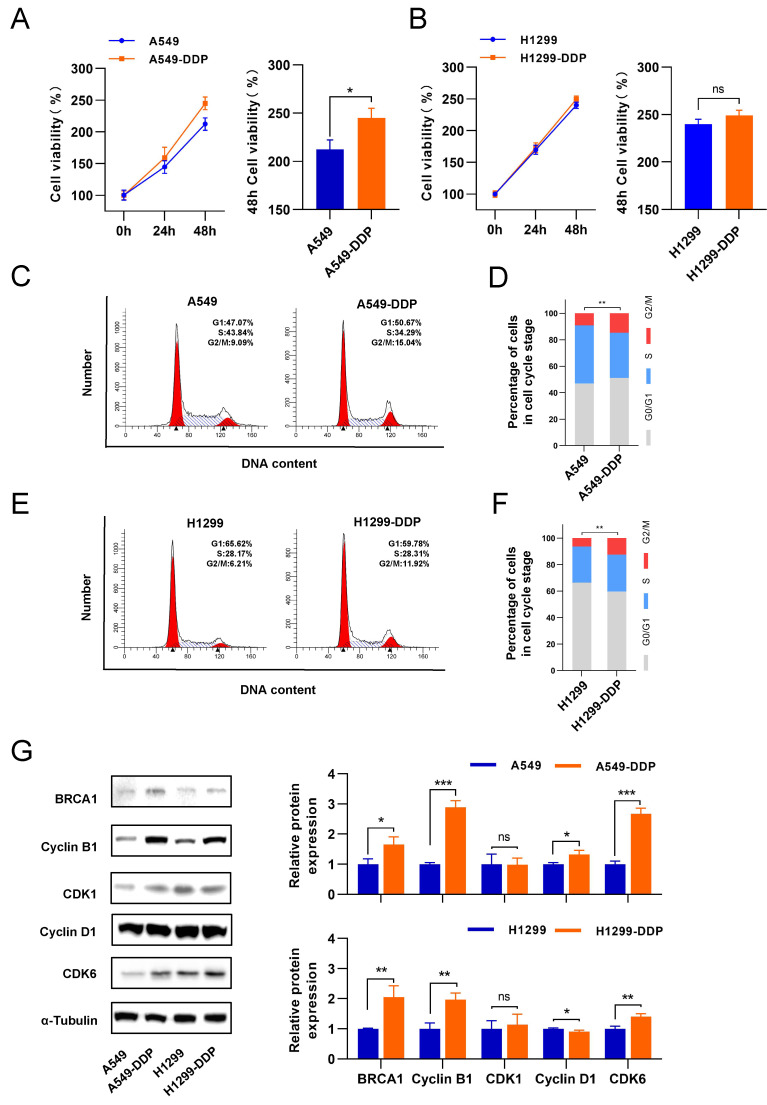
** G2/M phase of DDP-resistant LUAD was increased, accompanied by upregulation of cyclin B1 and BRCA1.** The proliferation of DDP-resistant LUAD cell lines and their parental cell lines, and the panels on the right showed the relative cell viability after 48 h **(A and B)**. Cell cycle analysis between DDP-resistant LUAD cell lines and their parental cell lines **(C-F)**. BRCA1 and cell cycle related protein were determined by western blotting **(G)**. Data are presented as the mean ± SEM. * *P* < 0.05, ** *P* < 0.01, *** *P* < 0.001, and ns, not significant.

Following the biological phenomenon we observed in CaSR-overexpressing cell lines, we investigated the changes in proliferation in LUAD cells before and after they acquired cisplatin resistance. We found that A549-DDP cells displayed slightly higher proliferation than A549 cells (Fig. 3A). Conversely, the proliferation levels of H1299 and H1299-DDP cells were similar (Fig. 3B). We thought that this difference occurred because of the inherent genomic alterations and molecular signature present in A549 (p53 wild-type) and H1299 (p53-deficient) cells, which are both lung adenocarcinoma cells 41, and the differential changes in the transcriptome after cisplatin treatment may also account for the different proliferation response. Next, the proportion of cells in the G2/M phase among DDP-resistant LUAD cells was significantly increased (Fig. 3C-F). Western blotting results showed that cyclin B1 and BRCA1 were upregulated in the DDP-resistant LUAD cells. In contrast, CDK1 did not show significant changes (Fig. 3G). Taken together with the previous findings in CaSR-overexpressing LUAD cells, we hypothesized that CaSR might affect the cisplatin resistance of LUAD cells through cyclin B1 and BRCA1.

### The cisplatin sensitivity in DDP-resistant LUAD cells was reversed by suppressing CaSR expression

**Figure 4:**
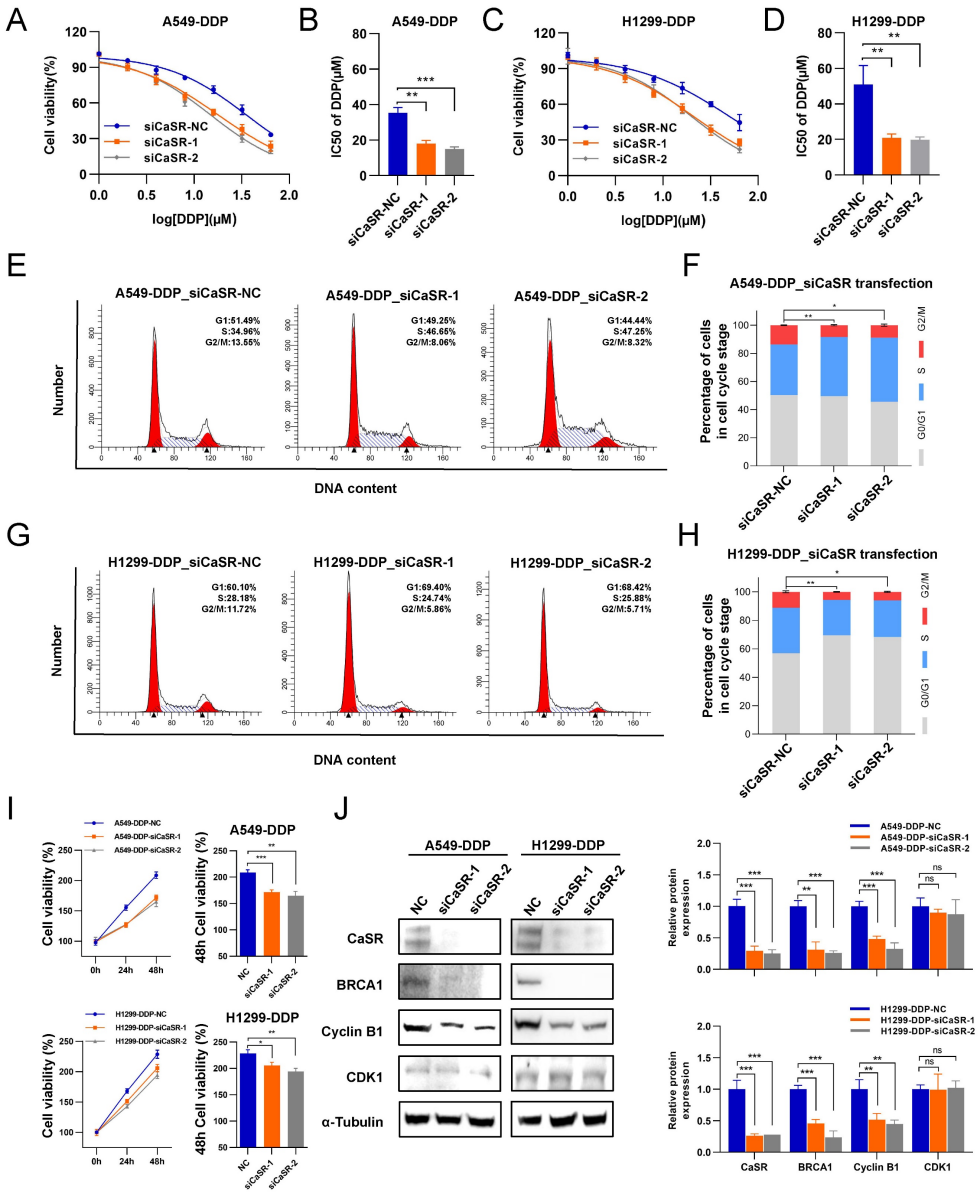
** CaSR was emerged as a potential target for reversing platinum-based chemotherapy resistance in LUAD.** A549-DDP and H1299-DDP cells were transfected with siRNA targeting CaSR (siCaSR-1, siCaSR-2) or non-targeting siRNA (siCaSR-NC), CaSR knockdown affected the sensitivity of A549-DDP cells **(A and B)** and H1299-DDP cells **(C and D)** to cisplatin. Cell cycle analysis showed that the G2/M phase was decreased after CaSR knockdown both in A549-DDP **(E and F)** and H1299-DDP cells (G and H).The proliferation ability of A549-DDP and H1299-DDP cells was impacted to a certain extent after CaSR knockdown and the right panels show the relative cell viability at the 48 h **(I)**. The protein levels of CaSR, cyclin B1, CDK1 and BRCA1 were determined by western blotting **(J)**. Data are presented as the mean ± SEM. * *P* < 0.05, ** *P* < 0.01, *** *P* < 0.001, **** *P* < 0.0001 and ns, not significant.

**Figure 5:**
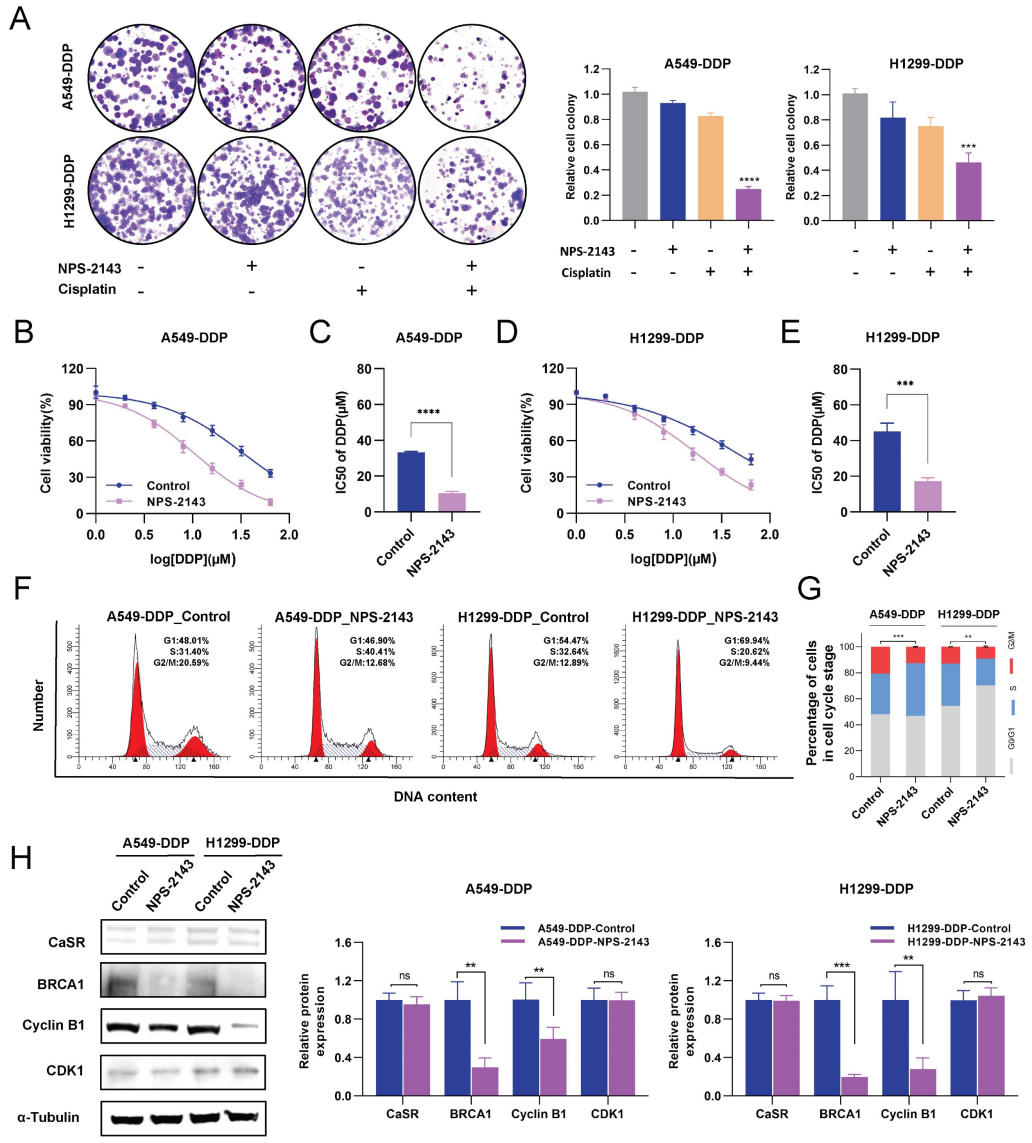
** NPS-2143 alleviated the cisplatin resistance of DDP-resistant LUAD cells.** The clonogenic proliferation of DDP-resistant cells was disrupted by the CaSR inhibitor NPS-2143 **(A)**. A549-DDP and H1299-DDP cells were treated with 10 µM cisplatin and 100 nM NPS-2143, respectively, or in combination. The sensitivity of A549-DDP **(B and C)** and H1299-DDP (D and E) cells to cisplatin was affected by the CaSR inhibitor NPS-2143. Cell cycle analysis showed that the changes after A549-DDP and H1299-DDP cells treated with 100 nM CaSR inhibitor NPS-2143 **(F and G)**. The protein levels of CaSR, BRCA1, cyclin B1, and CDK1 were detected after A549-DDP and H1299-DDP cells treated with 100 nM CaSR inhibitor NPS-2143 **(H)**. All datas are showed as the mean ± SEM. * *P* < 0.05, ** *P* < 0.01, *** *P* < 0.001, **** *P* < 0.0001 and ns, not significant.

To examine the necessity of CaSR for the acquisition of cisplatin resistance by DDP-resistant LUAD cell lines, we exploited small interfering RNAs (siRNAs) to disrupt CaSR expression in DDP-resistant cells. We found that DDP-resistant LUAD cells became more sensitive to cisplatin after CaSR knockdown (Fig. 4A-D). Furthermore, cell cycle analysis revealed that the proportion of cells in the G2/M phase was decreased after CaSR knockdown (Fig. 4E-H), which also suggested that the DNA damage repair response process was reduced. Cell proliferation assay revealed that the cell growth was affected after CaSR knockdown (Fig. 4I). Moreover, we detected decreased protein expression levels of BRCA1 and cyclin B1 (Fig. 4J). Combining multiple drugs to treat tumors or drug-resistant tumors is a common strategy in cancer treatment, and the current research into G protein-coupled receptor (GPCR)-targeted drugs is well advanced. Therefore, we examined the impact of CASR inhibitors on cisplatin resistance in lung cancer. The CaSR inhibitor NPS-2143 (Fig. S5A) was utilized in the following experiments. We noted that a higher concentration of NPS-2143 (1 µM) had a strong toxic effect on cell viability (Fig. S5B-D). Cisplatin combined with NPS-2143 (10 or 100 nM) was then administered. The clonogenic proliferation assay results revealed that the combination of 100 nM NPS-2143 with cisplatin (10 µM) suppressed the growth of DDP-resistant LUAD cells, and this effect was more pronounced in A549-DDP cells (Fig. 5A).

In contrast, the concomitant treatment consisting of 10 nM NPS-2143 did not considerably curtail the proliferation of DDP-resistant LUAD cells (Fig. S5B-D). We then investigated alterations in the cisplatin resistance of DDP-resistant LUAD cells following treatment with 100 nM NPS-2143 and observed that the cells exhibited increased cisplatin sensitivity (Fig. 5B-E), suggesting that a combination of NPS-2143 and cisplatin can mitigate the cisplatin resistance of DDP-resistant LUAD cells. The G2/M phase was similarly reduced following treatment with NPS-2143, as was observed after CaSR knockdown (Fig. 5F, G). Western blotting results showed that there was no notable distinction in CaSR expression after NPS-2143 treatment. Meanwhile, the protein expression levels of BRCA1 and cyclin B1 decreased (Fig. 5H), confirming our hypothesis and indicating that CaSR has effects on the closely related BRCA1 and the cell cycle and is involved in the development of cisplatin resistance in LUAD. Hence, CaSR was determined to be a potential target for reversing platinum-based chemotherapy resistance in LUAD.

### NPS-2143 combined with cisplatin significantly repressed the growth of DDP-resistant LUAD cells *in vivo*

**Figure 6:**
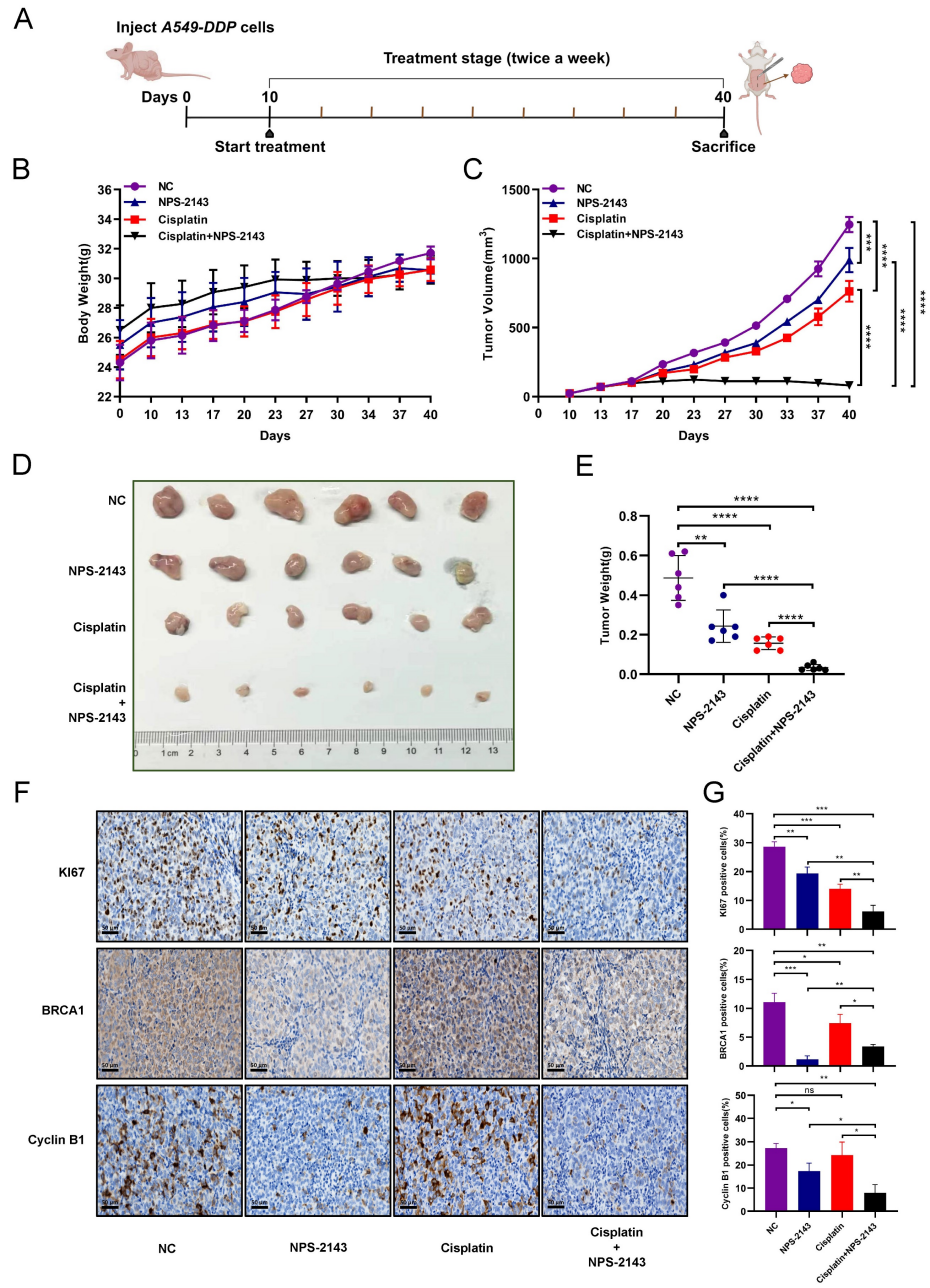
** NPS-2143 combined with cisplatin repressed the growth of DDP-resistant LUAD *in vivo*.** Schematic diagram of the experimental procedure in nude mice **(A)**. The A549-DDP cells were subcutaneously injected into nude mice as tumor xenografts. Four groups were treated with cisplatin (6 mg/kg), NPS-2143 (80 µg/kg), PBS and NPS-2143 in combination with cisplatin twice a week for one month, while body weight **(B)** and tumor **(C)** size were recorded. At the termination of the experiment, photographs of all tumors were taken **(D)**, and tumor weight was measured **(E)**. The expression of KI67, BRCA1 and cyclin B1 has been determined by IHC **(F)**, followed by statistics and corresponding images **(G)**. Data are presented as the mean ± SEM. Statistical significance was determined by one-way ANOVA with * *P* < 0.05, ** *P* < 0.01, *** *P* < 0.001, **** *P* < 0.0001 and ns, not significant.

NPS-2143, as a CaSR inhibitor, is commonly used in cancer research 42, 43. To evaluate the effect of the combination of a CaSR inhibitor and cisplatin on the growth of LUAD *in vivo*, the established A549-DDP cells were injected subcutaneously into nude mice as tumor xenografts. For approximately 1 month, each group received two weekly injections of PBS, cisplatin (6 mg/kg), NPS-2143 (80 µg/kg), and NPS-2143 (80 µg/kg) in combination with cisplatin (6 mg/kg). Mouse body weights and tumor sizes were recorded as shown in Fig. 6A. There were no significant differences in body weight among the groups before and after they were treated (Fig. 6B). In nude mice, tumor volume growth was significantly inhibited by the combined use of NPS-2143 and cisplatin (Fig. 6C). Correspondingly, the weights of the resected tumors in the drug combination groups were substantially lower than the other tumor weights (Fig. 6D, E). We subsequently conducted IHC assays on excised tumors from each group (Fig. 6F). As a marker for evaluating tumor proliferation, the reduction of KI67 confirmed that the tumor growth was repressed in the combination group. Moreover, in the NPS-2143 treatment group, the expression levels of BRCA1 and cyclin B1 were decreased, reflecting the effect of NPS-2143 to some extent. In the group that received only cisplatin treatment, the expression of BRCA1 was significantly higher compared to the NPS-2143 treatment group and the combination group, but slightly lower compared to the NC group. In comparison to the NC group, the cisplatin treatment group exhibited smaller tumor size (Fig. 6D) and slightly lower expression of BRCA1, indicating that cisplatin (6 mg/kg) had drug effect to a limited extent, but the inhibitory effect on the growth of DDP-resistant cells *in vivo* was not as effective as in the combination group. In the combination group, as expected, BRCA1 and cyclin B1 expression levels were significantly reduced (Fig. 6F, G). These results demonstrated a noteworthy decrease in the growth of DDP-resistant LUAD cells* in vivo* when treated with a combination of cisplatin and NPS-2143.

### KIF11 interacted with CaSR and was upregulated in DDP-resistant LUAD cells

**Figure 7:**
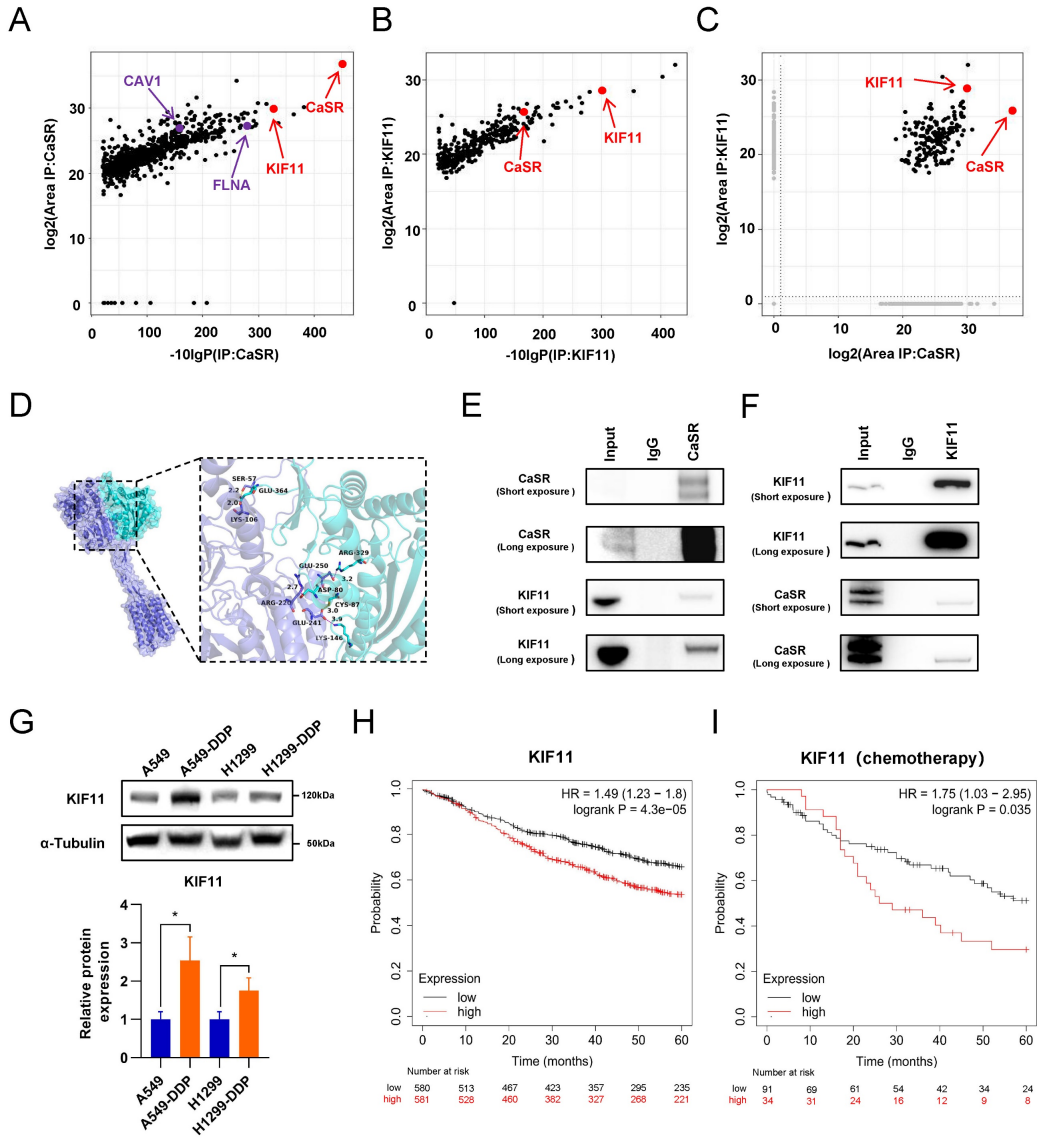
** KIF11 interacted with CaSR and was upregulated in DDP-resistant LUAD cells.** Mass spectrometry (MS) was used to identify proteins **(A-C)**, CaSR-interacting proteins **(A)**, KIF11-interacting proteins **(B)**, and proteins interacting with CaSR or KIF11 are selected based on two screening criteria: The protein was not pulled down by IgG or the protein had less than 2 folds increase in detection in IgG compared to CaSR or KIF11 **(C)**. Protein-protein interaction maps between CaSR and KIF11 **(D)**, CaSR is depicted in dark blue, and KIF11 is depicted in cyan. The corresponding binding points are indicated as clubbed structures of the corresponding colors. And western blotting results showed clear detection of KIF11 in the CaSR-interacting proteins **(E)** and CaSR in the KIF11-interacting proteins **(F)**. KIF11 was highly expressed in LUAD **(G)**. Kaplan-Meier plots showed the correlation of KIF11 with overall survival in LUAD **(H)**, including the patient underwent chemotherapy **(I)**.

The results of the study described above indicated that the impact of CaSR on cisplatin sensitivity in LUAD may be related to BRCA1 and cyclin B1. However, a direct relationship between CaSR, BRCA1, and cyclin B1 has not yet been well documented. Moreover, how CaSR impacts the repair of DNA damage and the cell cycle is a topic of significant interest. To explore this question, we performed co-immunoprecipitation (coIP) assessments of protein lysates from the A549-DDP cell line using CaSR antibodies to identify potential CaSR-interacting proteins by mass spectrometry (MS). The top 20 scoring proteins in the identification results are listed in Table S3. Among them, filamin A (FLNA) and caveolin 1 (CAV1), which are known to interact with CaSR 44, 45, were shown as reference proteins for successful coIP and MS analyses (Fig. 7A). Considering that BRCA1 and cyclin B1 were found to be affected by CaSR and were associated with the cell cycle and DNA damage repair in the above studies, we selected proteins associated with DNA damage repair or mitosis from the CaSR-interacting proteins. Surprisingly, KIF11, a protein that plays key roles in mitosis and the cell cycle 46, was identified. KIF11-interacting proteins were also identified by MS, and CaSR was among the identified proteins (Fig. 7B, C). The protein identification results that scored in the top 20 are listed in Table S4. Next, molecular docking prediction of CaSR and KIF11 was performed. We found that CaSR and KIF11 have multiple predictable binding sites, most involving ionic bonds (Fig. 7D). We then investigated the interaction between CaSR and KIF11 through coIP assessments to identify interactions with either CaSR or KIF11. KIF11 was clearly detected among the CaSR-interacting proteins (Fig. 7E); likewise, CaSR was detected among the KIF11-interacting proteins (Fig. 7F). KIF11 was found to be highly expressed in various types of malignant tumors, including LUAD (Fig. S6A, B). Upon further investigation, we found that KIF11 expression was higher in DDP-resistant LUAD than in parental cells (Fig. 7G), which appeared to be related to the high expression of CaSR in DDP-resistant LUAD cells. Moreover, KIF11 exhibited a poor correlation with CaSR but strong and positive correlations with BRCA1 and cyclin B1 (Fig. S6C). Kaplan-Meier plotter server analysis also revealed an association between KIF11 and the survival and prognosis of patients with LUAD (Fig. 7H) Importantly, high expression of KIF11 was found to be significantly associated with poor prognosis in LUAD with chemotherapy (Fig. 7I).

### KIF11 acted as a crucial mediator of cisplatin resistance in LUAD induced by CaSR

**Figure 8:**
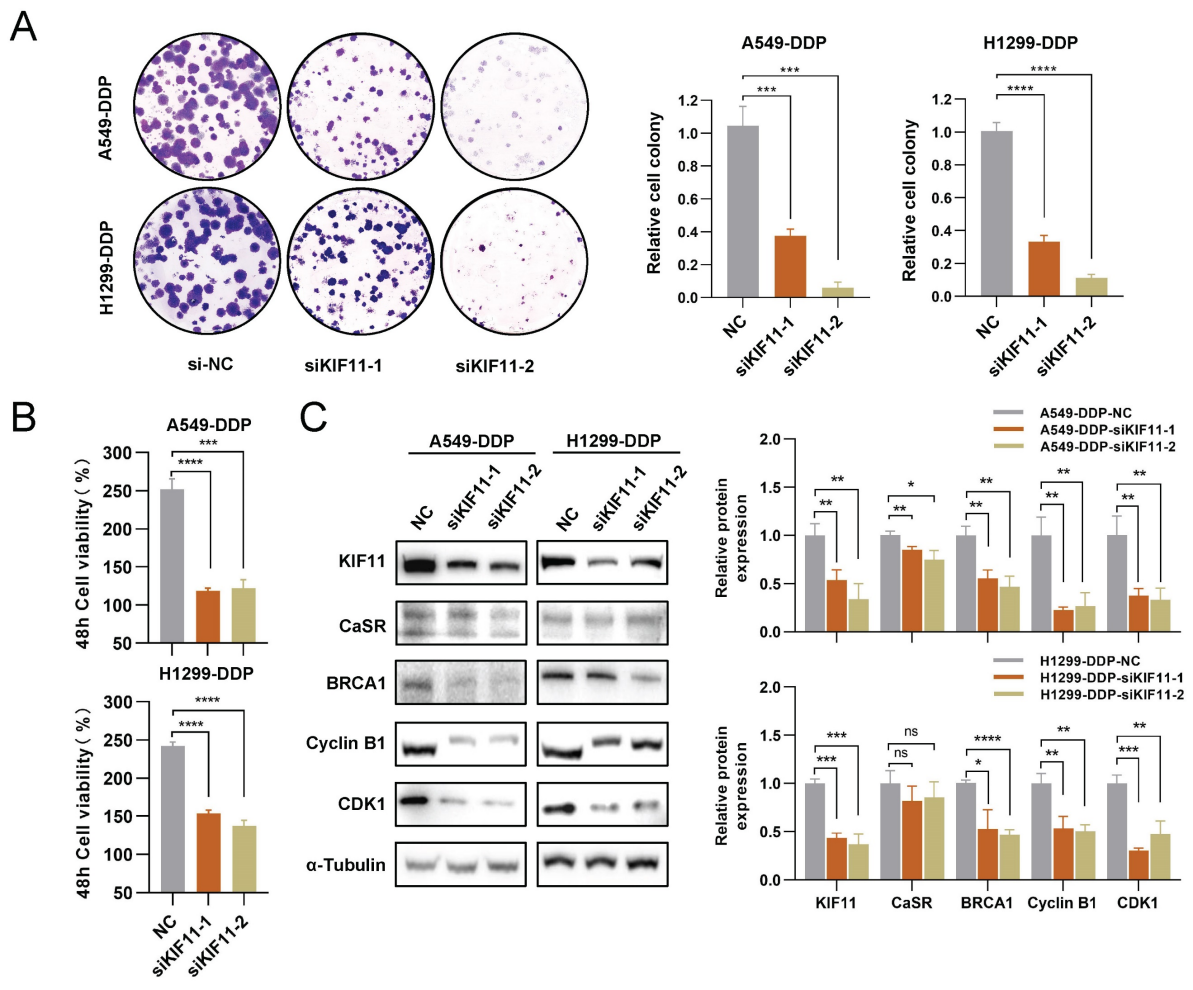
** Downregulation of KIF11 inhibited the growth of DDP-resistant LUAD cells.** A549-DDP and H1299-DDP cells were transfected with siRNA targeting KIF11 (siKIF11-1, siKIF11-2) or non-targeting siRNA (si-NC). After KIF11 knockdown, the clonogenic proliferation of cisplatin-resistant cells was disrupted **(A)**. The proliferation ability of A549-DDP and H1299-DDP were inhibited and the panels show the relative cell viability at the 48 h **(B)**. Western blotting results showed the changes on protein levels of KIF11, CaSR, cyclin B1, CDK1 and BRCA1 **(C)**. Data are presented as the mean ± SEM. * *P* < 0.05, ** *P* < 0.01, *** *P* < 0.001, **** *P* < 0.0001 and ns, not significant.

The above investigations revealed that KIF11 exhibited a close association with the prognosis of LUAD under chemotherapy and interacted with CaSR through protein-protein binding. We then investigated the role of KIF11 in CaSR-mediated cisplatin resistance in LUAD cells by disrupting KIF11 expression in DDP-resistant LUAD cells using siRNA transfection. Clonogenicity assay results further demonstrated that KIF11 knockdown significantly inhibited cell growth (Fig. 8A) and cell proliferation assay revealed that the cell growth was almost arrested following KIF11 knockdown (Fig. 8B). The inhibition of cell growth made it challenging to observe the alterations in cisplatin sensitivity of DDP-resistant LUAD cells after KIF11 knockdown, as determined by CCK8 assay. Considering the previous speculation that KIF11 may be the mediator by which CaSR affects BRCA1 and cyclin B1, we further examined the expression levels of BRCA1 and cyclin B1 in DDP-resistant LUAD cells after KIF11 knockdown and observed significant decreases in the expression levels of BRCA1 and cyclin B1 after KIF11 knockdown (Fig. 8C). Furthermore, we investigated the impact of KIF11 knockdown on CaSR and observed a slight reduction in A549-DDP cells (Fig. 8C). However, we did not observe a significant effect of KIF11 knockdown on CaSR expression in H1299-DDP cells. Additionally, CDK1 expression in DDP-resistant LUAD cells was significantly inhibited after KIF11 knockdown, suggesting that KIF11 knockdown may affect more aspects of the cell cycle.

**Figure 9:**
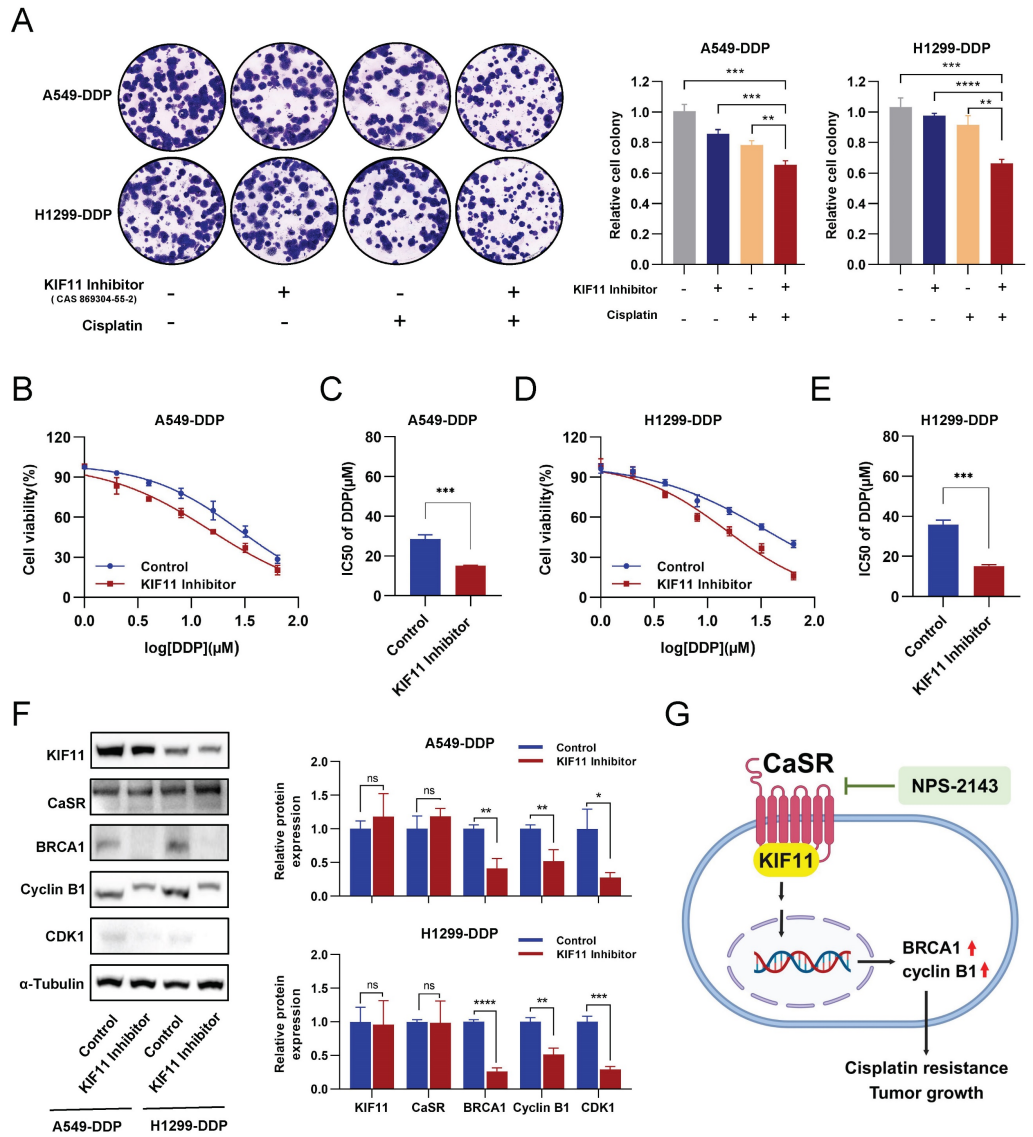
** Inhibition of KIF11 reduced cisplatin resistance in DDP-resistant LUAD cells.** The clonogenic proliferation of DDP-resistant cells was disrupted by the KIF11 inhibitor **(A)**. A549-DDP cells and H1299-DDP cells were treated with 10 µM cisplatin and 1µM KIF11 inhibitor, respectively, or in combination. Colony formation assay was performed to analyze the cells, and the right panel quantifies the relative number of colonies formed after 14 days. The sensitivity of A549-DDP **(B and C)** and H1299-DDP **(D and E)** cells to cisplatin was affected by the KIF11 inhibitor. The protein levels of KIF11, CaSR, cyclin B1, CDK1 and BRCA1 were detected by western blotting **(F)**. The schematics illustrate the proposed mechanism of CaSR activates the cisplatin resistance in LUAD **(G)**. Data are showed as the mean ± SEM. * *P* < 0.05, ** *P* < 0.01, *** *P* < 0.001, **** *P* < 0.0001 and ns, not significant.

Considering that KIF11 knockdown has a strong effect on cell growth, we then employed KIF11 inhibitors commonly used in cancer research (Fig. S7A). Similarly to KIF11 knockdown, the growth levels of A549-DDP and H1299-DDP cells treated with 10 µM of KIF11 inhibitor were significantly inhibited or even negative (Fig. S7B-D), though the growth inhibition of DDP-resistant LUAD cells receiving KIF11 inhibitor (1 µM) was not obvious (Fig. S7C-D). The clonogenic proliferation assay demonstrated that either the KIF11 inhibitor (1 µM) or cisplatin (10 µM) alone was not significantly effective in inhibiting or eliminating DDP-resistant LUAD cells. In contrast, the combination of the KIF11 inhibitor and cisplatin significantly inhibited the growth of LUAD cells (Fig. 9A). Importantly, similar to treatment with the CaSR inhibitor NPS-2143, DDP-resistant LUAD cells treated with the KIF11 inhibitor (1 µM) showed increased sensitivity to cisplatin (Fig. 9B-E). Western blotting results revealed that treatment with the KIF11 inhibitor (1 µM) did not have an impact on the expression of KIF11 or CaSR in DDP-resistant LUAD cells. Notably, treatment with the KIF11 inhibitor significantly decreased the expression levels of BRCA1 and cyclin B1 (Fig. 9F). To verify the causal relationship, we performed knockdown of BRCA1 in A549-DDP and H1299-DDP cells, observed a significant reduction in cyclin B1 expression upon BRCA1 knockdown. Previous studies have reported the involvement of BRCA1 in the expression and stability of cyclin B1 in breast cancer 40, 47. Meanwhile, the knockdown of BRCA1 did not result in significant changes in CaSR and KIF11 expression in A549-DDP and H1299-DDP cells (Fig. S8). Based on the observed changes in the sensitivity of DDP-resistant LUAD cell lines to cisplatin after treatment with the KIF11 inhibitor, as well as the detected alterations in the protein levels of CaSR, BRCA1, and cyclin B1, we suggest that the interaction between CaSR and the KIF11 protein could potentially play a role in the pathway through which CaSR influences cisplatin resistance in LUAD cells.

## Discussion

Lung cancer is the leading cause of cancer-related deaths worldwide and is proving to be a challenging disease to treat. Cisplatin, one of the principal first-choice treatments for NSCLC, efficiently inhibits tumor growth, contributes to the management of the condition, and yields remarkable therapeutic outcomes 48, 49. However, the development of chemoresistance often leads to treatment failure. Overcoming resistance to cisplatin and enhancing its efficacy continue to be pressing issues in clinical practice. In this study, we analyzed the transcriptomic changes between lung adenocarcinoma cell lines before and after acquiring cisplatin resistance and determined that CaSR was significantly upregulated in DDP-resistant LUAD. The outcome of public database comparisons provided initial evidence that patients with LUAD and clinically elevated levels of CaSR expression were prone to a distressing prognosis following chemotherapy, suggesting that CaSR may play an influential role in the development of tumor resistance to chemotherapy.

CaSR has emerged as an important receptor to target in the treatment of cancer due to the important functions it plays in tumors 50. In this study, we found that LUAD cell lines overexpressing CaSR had decreased sensitivity to cisplatin compared to the parental cell lines and that platinum drug resistance and cell cycle-related pathways were upregulated at the transcriptional level in the two LUAD cell lines overexpressing CaSR. Simultaneously, pathways related to DNA damage repair were also activated. It is known that cisplatin hinders tumor growth by affecting DNA replication and repair mechanisms within tumor cells 51. The activation of these pathways suggests that CaSR may function in mediating cisplatin resistance. Further investigation revealed that both DDP-resistant and CaSR-overexpressing LUAD cells exhibited significant increases in cells in the G2/M phase by cell cycle analysis. We know that normal cells typically prolong the G1 phase of cell division during the DNA repair process. However, most tumor cells have a dysfunctional G1 checkpoint and often require a functional G2/M checkpoint for DNA repair 52, 53. Combined with our results, it is reasonable to speculate that the prolongation of the G2/M phase of these cells gives them more time to self-repair and acquire resistance to cisplatin. We also observed a significant upregulation of cyclin B1, a protein closely associated with the G2/M phase of the cell cycle and chemoresistance. Several studies have demonstrated the crucial involvement of cyclin B1 in promoting cell cycle progression and enhancing the development of drug-resistant tumors. Additionally, researchers have found that cyclin B1 could be linked to the metabolic reprogramming involved in tumor adaptive resistance 54, 55. Recent studies have indicated that disruption of cyclin B1 can affect the sensitivity of nasopharyngeal carcinoma cells to cisplatin 56. Meanwhile, high levels of BRCA1 have been detected in LUAD cell lines that are resistant to cisplatin. BRCA1 was the first gene linked to hereditary breast cancer [57]and acts as a tumor suppressor with several functional domains involved in significant biological processes, such as DNA damage repair and regulation of the G2/M checkpoint 58. BRCA1 has also been implicated in the response to cisplatin resistance. Platinum-induced DNA cross-linking, a significant and lethal form of DNA damage that can lead to double-stranded breaks (DSBs), is well established, and BRCA1 is important in activating DSB repair 59. According to an analysis of prognostic data from 57 patients with locally advanced bladder cancer, patients with elevated BRCA1 expression were less likely to benefit from cisplatin-based therapy 60. In addition, under conditions of DNA damage, BRCA1 can increase the stability of cyclin B1 through protein interactions 40. In NSCLC cells that are resistant to cisplatin, the homologous recombination repair function of BRCA1 has been reported to be enhanced 61, supporting our findings to some extent. Therefore, we hypothesize that CaSR may affect the cisplatin resistance of tumor cells through its effects on cyclin B1 and BRCA1.

To comprehensively assess the role of CaSR, siRNA interference was employed to suppress CaSR expression in DDP-resistant LUAD cells. The sensitivity to cisplatin in DDP-resistant LUAD cells was increased after CaSR knockdown, and the protein expression levels of BRCA1 and cyclin B1 were decreased. We observed that overexpression and knockdown of CaSR all influenced the proportion of cells in the G2/M phase, but leading to varied effects on cell proliferation. As we know, cell proliferation is a multifaceted process influenced by factors such as cell cycle, metabolism and apoptosis 62-64. Relying solely on cell cycle distribution may not accurately determine the cell's proliferation. Furthermore, we detected the effects of CaSR overexpression on the metabolism and apoptosis of LUAD cells. The overexpression of CaSR in LUAD cells resulted in a reduction in glycolysis and glycolytic capacity (Fig. S4F). Meanwhile, the percentage of apoptosis cells was significantly decreased in LUAD cells overexpressing CaSR (Fig. S4G). Given these findings, it is plausible that the combined effects of these conditions influence the growth status of LUAD cells.

CaSR modulators have various potential applications in treating certain diseases 65. To further confirm the role of CaSR, we also performed *in vitro* and *in vivo* experiments with CaSR inhibitors. The results showed that NPS-2143 could reverse the resistance of DDP-resistant LUAD cells *in vitro*. In our subsequent experiment, cisplatin combined with NPS-2143 was found to significantly suppress the growth of DDP-resistant LUAD cells in immunodeficient mice. These findings provide recommendations for overcoming cisplatin resistance in LUAD. In addition, both *in vitro* and *in vivo*, NPS-2143 showed an effect on the protein levels of BRCA1 and cyclin B1 in DDP-resistant LUAD cells.

Currently, little is known about the relationship between CaSR, BRCA1, and cyclin B1. It has been reported that the administration of the CaSR agonist NPS-R-568 in oocytes can increase the levels of cyclin B1 66, while CaSR can protect BRCA1-deficient cells from the negative consequences of the loss of BRCA1 function 67. However, the effects of CaSR on BRCA1 and cyclin B1 have not been previously evaluated. To or knowledge, this was the first study to propose a protein interaction between CaSR and KIF11. Specifically, KIF11 is known to play a critical role in mitosis and is closely related to the G2/M phase 68. We also found that KIF11 was positively correlated with BRCA1 and cyclin B1 expression in the LUAD gene co-expression database. In addition, KIF11 knockdown or inhibition was observed to decrease the protein expression levels of BRCA1 and cyclin B1 but did not significantly affect the protein expression of CaSR. Meanwhile, depletion of BRCA1 resulted in a significant decrease in cyclin B1 levels, but did not have a significant effect on the expression of CaSR and KIF11 in A549-DDP and H1299-DDP cells. These findings indicate that KIF11 serves as a crucial mediator of CaSR, which can influence cisplatin resistance as well as the expression of BRCA1 and cyclin B1 in LUAD cells. Furthermore, suppression of KIF11 led to a reduction in CDK1 expression and induced substantial cell growth arrest. These findings suggest that the role of KIF11 may be multifaceted and should be taken into consideration. Further studies are needed to determine how CaSR and KIF11 interact to influence their respective functions, how KIF11 influences the expression of BRCA1 and cyclin B1, and whether there are alternative pathways by which CaSR contributes to DNA damage repair and mitosis.

## Conclusion

In this study, we identified a novel mechanism of cisplatin resistance in LUAD cells (Fig. 9G). CaSR enhanced cisplatin resistance in LUAD cells by affecting BRCA1 and cyclin B1, through KIF11 interaction. Moreover, CaSR altered the ability of LUAD to combat DNA damage and comprehensively changed the sensitivity of LUAD to cisplatin. Hence, we presented a novel molecular marker for diagnosing chemosensitivity in LUAD and a potential method for reversing multidrug resistance. Our study also suggested that the combination of a CaSR inhibitor and cisplatin may represent a new therapeutic strategy for the treatment of chemoresistant LUAD.

## Supplementary Material

Supplementary figures and tables.
