# Supplementary material for: The Interaction of Calcium-Sensing Receptor with KIF11 Enhances Cisplatin Resistance in Lung Adenocarcinoma via BRCA1/cyclin B1 pathway
Source: Int J Biol Sci. 2024 Jul 15;20(10):3892–910. doi: 10.7150/ijbs.92046 (PMC11302892; doi:10.7150/ijbs.92046)
Supplement: Supplementary file 1 — Supplementary figures and tables. [file ijbsv20p3892s1.pdf]

1. Supplementary figtures

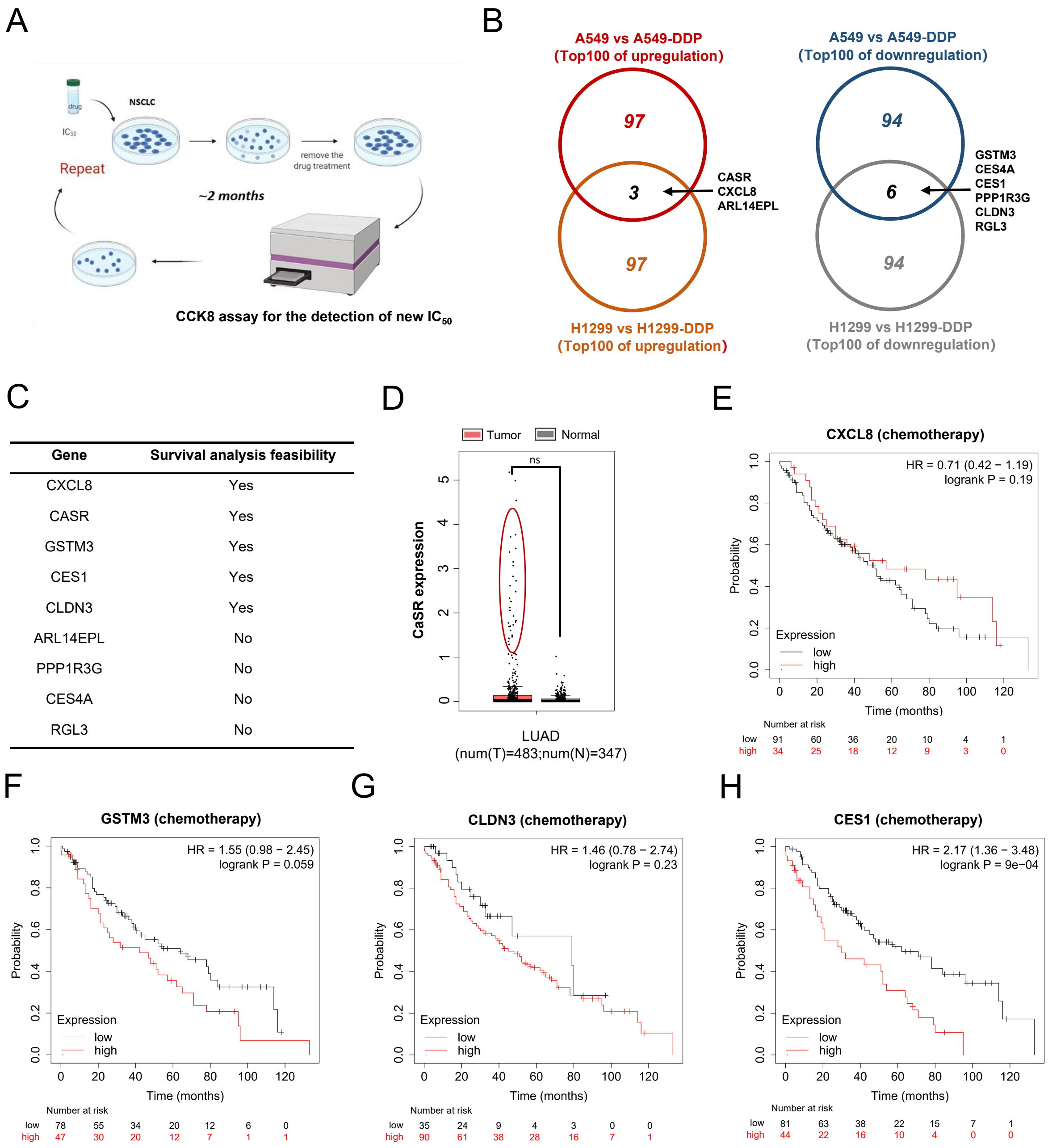

Figure S1. Establishment of DDP-resistant LUAD cells and the application of Kaplan-Meier plots to evaluate the prognosis post-chemotherapy in LUAD. Schematic diagram of the process of establishing DDP-resistant tumour cells (A).Venn diagram showed the overlap of differentially detected genes(B).The survival analysis feasibility of interested genes. Due to insufficient expression data, some genes cannot be conducted for the overall survival analysis of LUAD pateints receiving chemotherapy (C). CaSR expression in LUAD (D). Kaplan-Meier plots showed the correlation of CXCL8 (E), GSTM3 (F), CLDN3 (G) and CES1 (H) with overall survival in LUAD receiving chemotherapy.

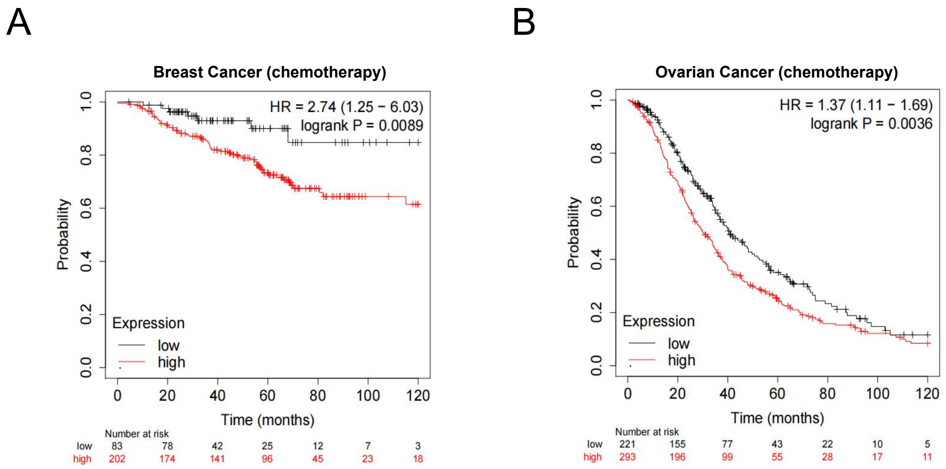

Figure S2. High expression of CaSR was associated with a worse prognosis following chemotherapy in breast cancer patients who did not receive endocrine therapy (A) and in ovarian cancer patients who underwent suboptimal debulking surgery (B).

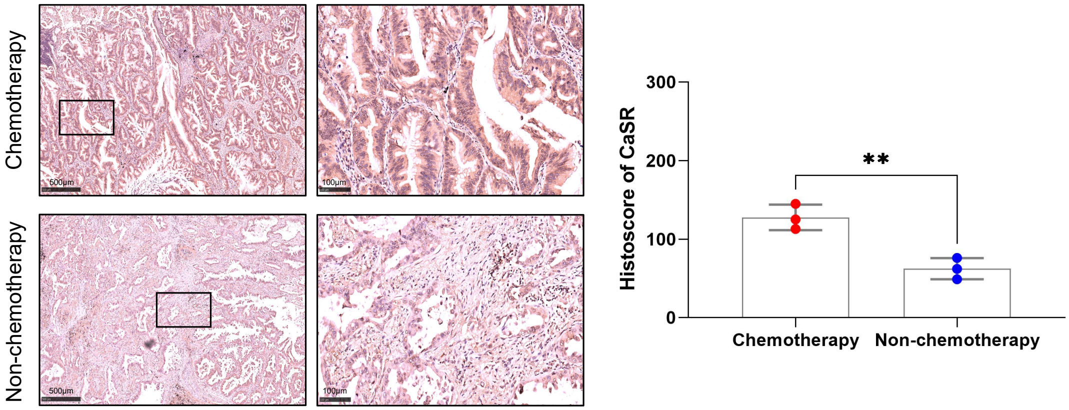

Sample	Cancer	Tumor staging	Treament	IHC staining of CaSR	Histoscore units
C1	LUAD	Phase II	Chemotherapy	Positive	135 ± 9
C2	LUAD	Phase III	Chemotherapy	Positive	125 ± 6
C3	LUAD	Phase II	Chemotherapy	Positive	113 ± 8
C4	LUAD	Phase III	Non-chemotherapy	Low positive	49 ± 12
C5	LUAD	Phase II-III	Non-chemotherapy	Low positive	76 ± 9
C6	LUAD	Phase II	Non-chemotherapy	Low positive	62 ± 3

Figure S3. CASR expression was evaluated using IHC in clinical samples from LUAD patients who either received or did not receive chemotherapy. The histoscore units were calculated as a percentage of different positive cells using the formula (3+)×3+(2+)×2+(1+)×1 by IHC-Profilter. Data are presented as the mean ± SEM. * $P < 0.05$, ** $P < 0.01$ and ns, not significant.

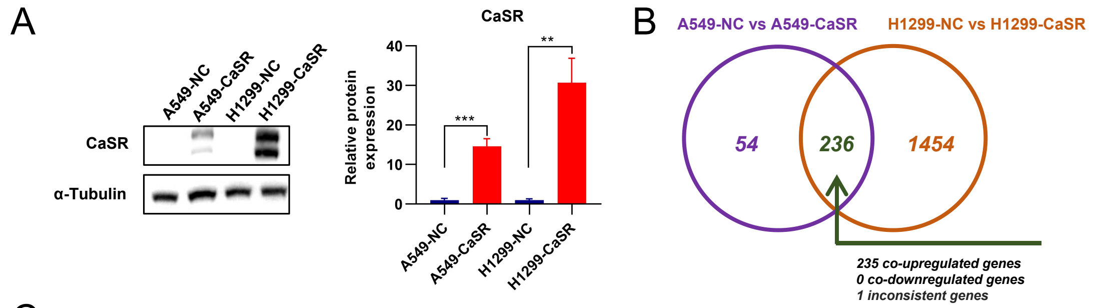

C

Category	Signaling pathways	Description	Genes
KEGG Pathway	hsa01524	*Platinum drug resistance*	*BRCA1 / MSH6 / MDM2 / MAP3K5 / MSH3 / REV3L / TOP2A*

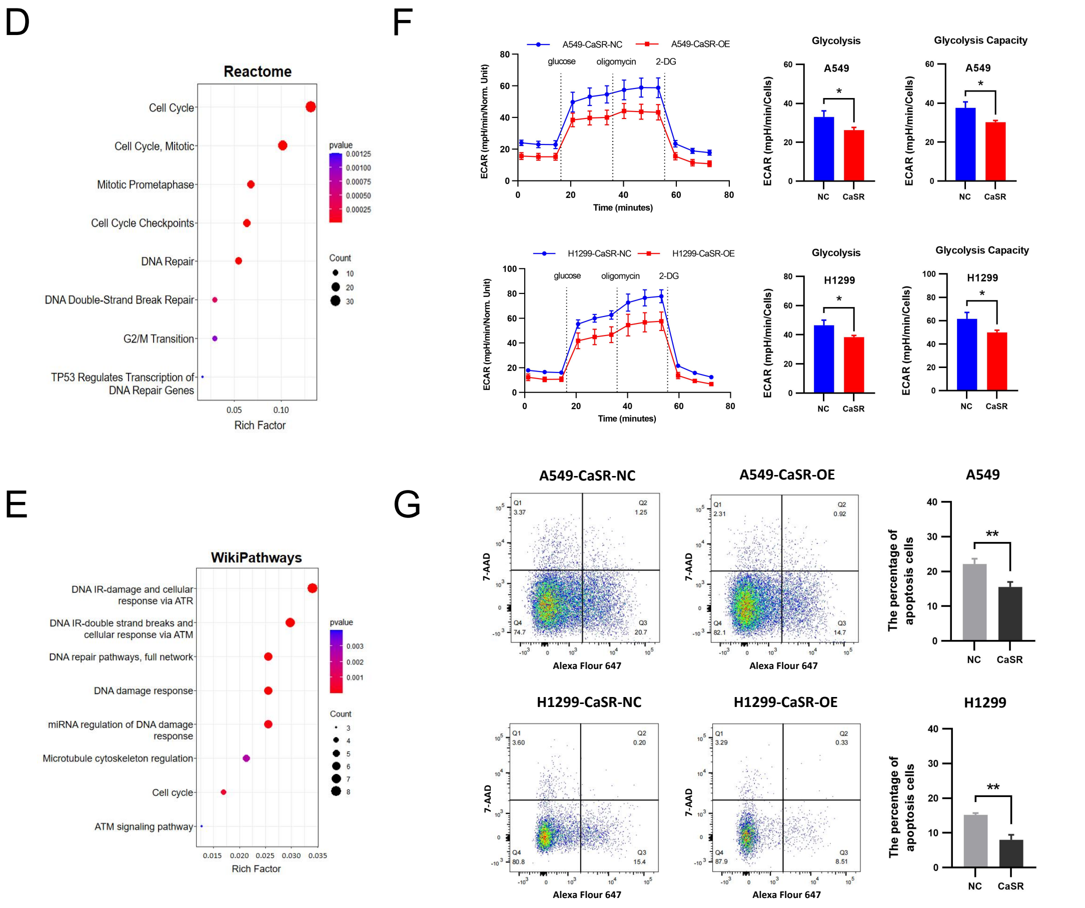

Figure S4. Overexpression of CaSR affected the cell cycle and cisplatin resistance in LUAD cell lines. Western blotting analysis showed the CaSR-overexpressing stable cell line was established in A549 and H1299 cells (A). Venn diagram showed the overlap of differentially detected genes between CaSR-overexpressing cell lines and negative control cell lines (B). The details of the KEGG (hsa01524) enrichment genes (C). Reactome (D) and WikiPathways (E) enrichment analysis. Overexpression of CaSR affected the glycolysis (F) and apoptosis (G) in LUAD cell lines. Data are presented as the mean ± SEM. * $P < 0.05$, ** $P < 0.01$ and ns, not significant.

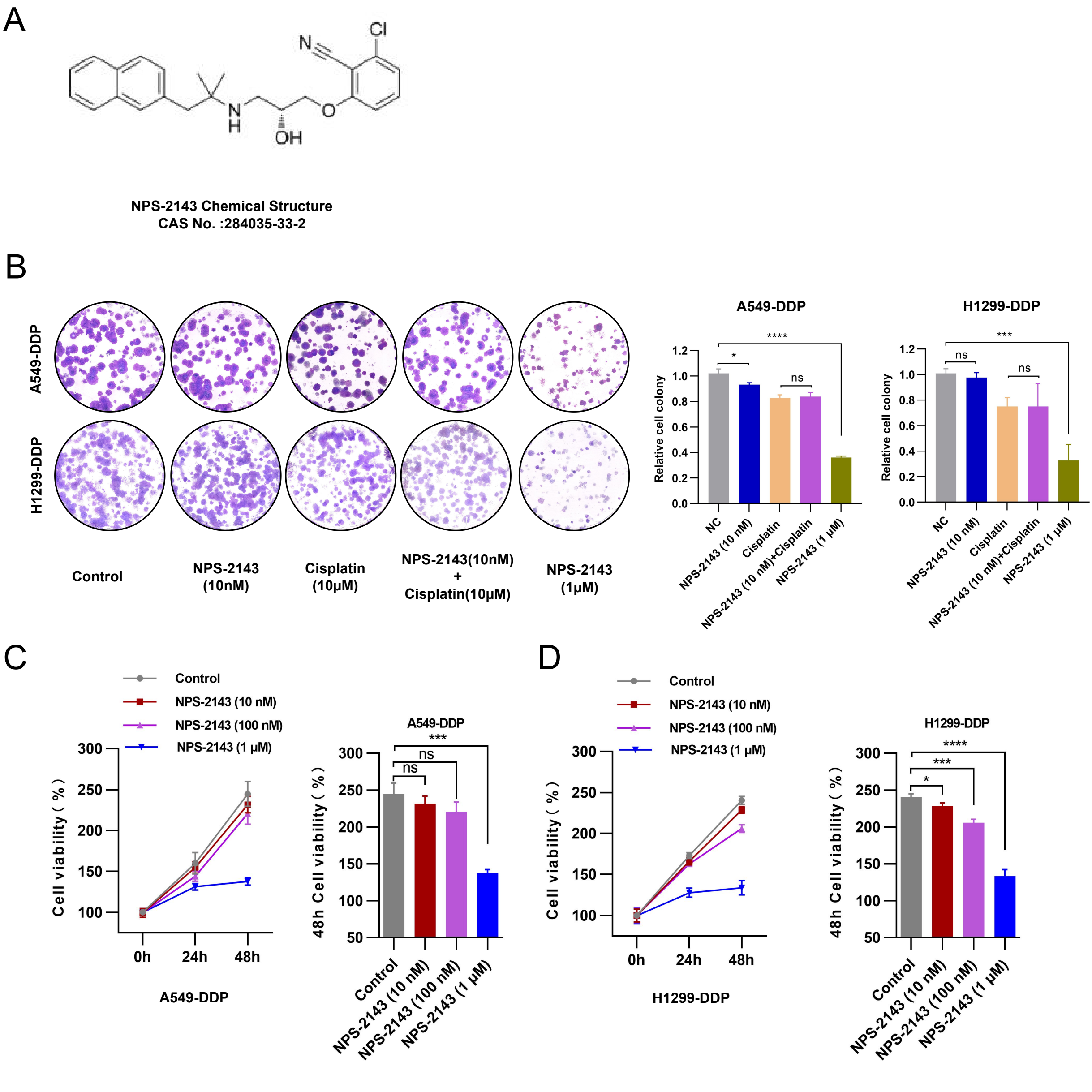

Figure S5. Effects on DDP-resistant LUAD cells of different concentrations of NPS-2143. Chemical structure of NPS-2143(A).The clonogenic proliferation of DDP-resistant cells. 1 μM NPS-2143 had clearly harmful and adverse effects both in A549-DDP and H1299-DDP cells. A549-DDP cells and H1299-DDP exhibited no significant inhibition or cytotoxicity that treated with 10 μM cisplatin and 10 nM NPS-2143, respectively, or in combination. The cells were analyzed through the colony formation assay, and the relative number of colonies formed after 14 days was quantified in the right panel (B). The proliferation ability of A549-DDP (C) and H1299-DDP (D) was affected upon exposure to varying concentrations of the NPS-2143 and the right-hand panels indicate the relative cell viability at 48 h. All datas are showed as the mean ± SEM. * $P < 0.05$, ** $P < 0.01$, *** $P < 0.001$, **** $P < 0.0001$ and ns, not significant.

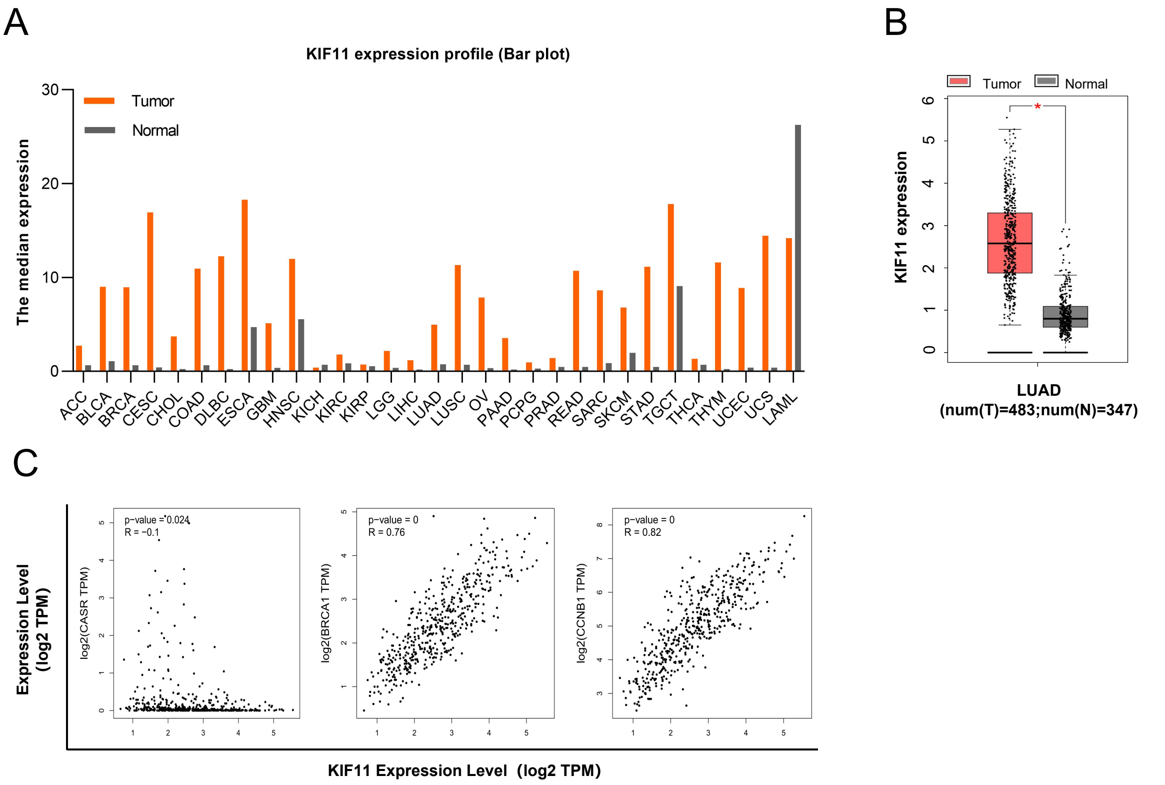

Figure S6. KIF11 acted as a crucial mediator of cisplatin resistance in LUAD induced by CaSR. KIF11 was highly expressed in various types of malignant tumours (A). KIF11 was upregulated in LUAD cells (B). Gene co-expression analysis of KIF11 with CaSR, BRCA1, and cyclin B1 (C)

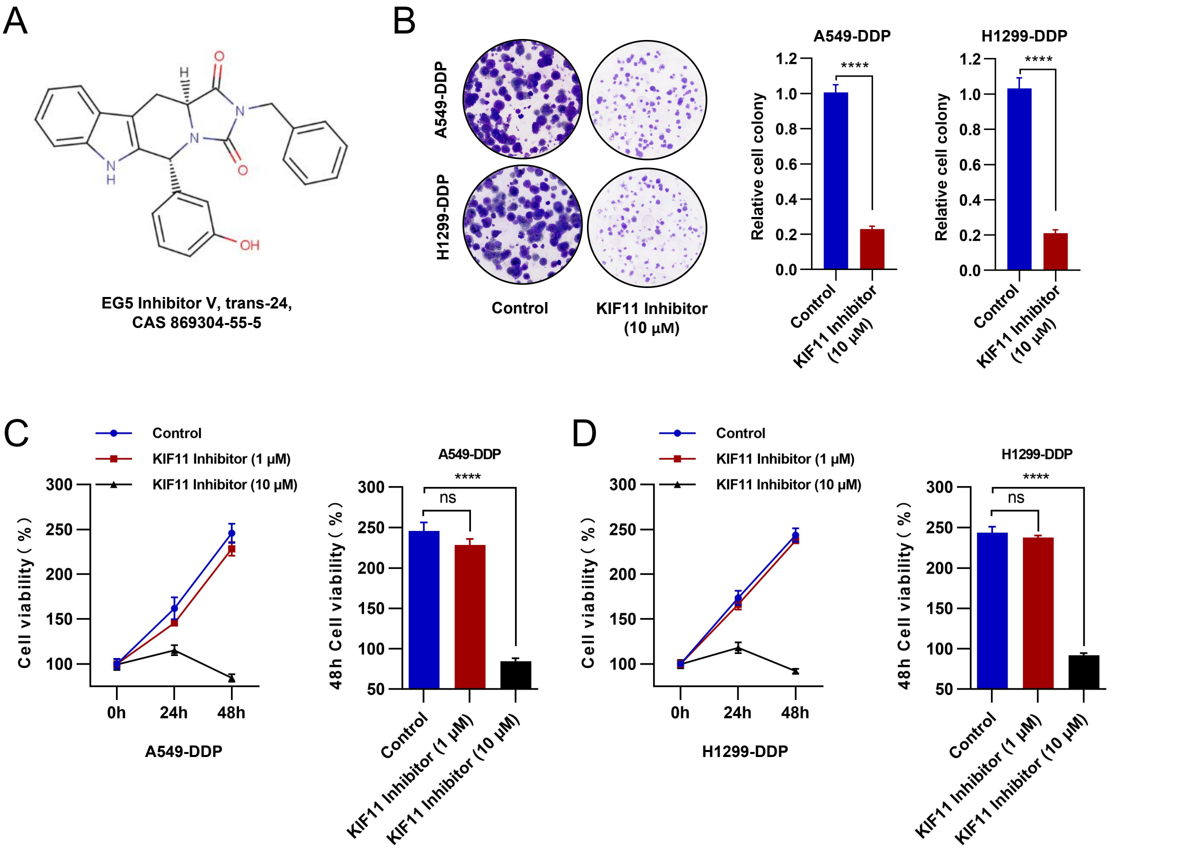

Figure S7. The impact of KIF11 inhibitor treatment on DDP-resistant LUAD cells. Chemical structure of KIF11 inhibitor (A). The clonogenic proliferation of DDP-resistant cells (B). 10 μM KIF11 inhibitor had clearly adverse effects both in A549-DDP and H1299-DDP cells. And the proliferation ability of A549-DDP (C) and H1299-DDP (D) was impacted to a certain extent treated with different concentrations of KIF11 inhibitor and the right panels show the relative cell viability at the 48 h. All datas are showed as the mean ± SEM. * $P < 0.05$, ** $P < 0.01$, *** $P < 0.001$, **** $P < 0.0001$ and ns, not significant.

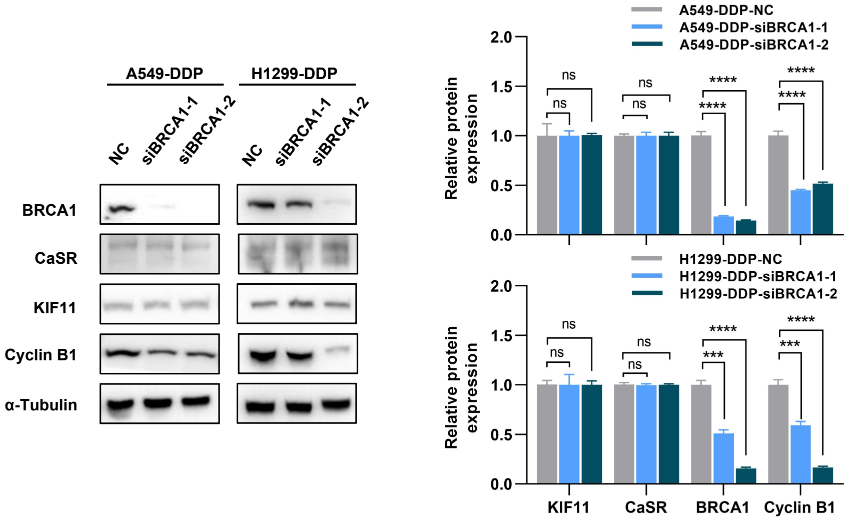

Figure S8. Effects on DDP-resistant LUAD cells after downregulated of BRCA1.

2. Supplementary tables

Table S1: Antibodies used in this study

Antibody	Company	Cat#	Dilution	Assay
CaSR	Cell Signaling Technology	73303S	1:1000	WB
BRCA1	Cell Signaling Technology	9010S	1:1000	WB
Cyclin B1	GENXSPAN	GXP73161	1:1000	WB
CDK1	GENXSPAN	GXP95882	1:1000	WB
Cyclin D1	Cell Signaling Technology	2978S	1:1000	WB
CDK6	Cell Signaling Technology	13331S	1:1000	WB
KIF11 (EG5)	GENXSPAN	GXP284254	1:1000	WB
HRP-conjugated Tubulin	Proteintech	HRP-66031	1:5000	WB
CaSR	Abcam	ab19347	1：200	IHC
BRCA1	Protrintech	22362-1-AP	1:800	IHC
Cyclin B1	GENXSPAN	GXP73161	1:300	IHC
KI67	Abcam	ab15580	1:400	IHC
KIF11 (EG5)	GENXSPAN	GXP284254	1:100	IP
CaSR	Cell Signaling Technology	73303S	1:100	IP

Table S2: The targeting sequences of siRNA used in this study

siRNA name	Sequence
Non-targeting siRNA	ACGUGACACGUUCGGAGAATT
siCaSR-1	CGCCUUGCAAGAUAUAUAUTT
siCaSR-2	GAGAGGAAGCUGAGGAAAGTT
siKIF11-1	CGAAGAAGAAAGAGGAGAATT
siKIF11-2	GGAAAGUACUGAGGAGAAATT
siBRCA1-1	GAUCAAGAAUUGUUACAAATT
siBRCA1-2	GAGUAAUAUUGAAGACAAATT

Table S3: Top 20 candidates of CaSR-interacting proteins.

Protein name	Accession	Score[1]	Coverage[2]	Peptides[3]	Subcellular localization
**CASR**	P41180	449.75	62	174	Cell membrane
PLEC	Q15149	364.37	26	109	Cytoplasm, cytoskeleton
MYH10	P35580	335.73	35	62	Cell projection
**KIF11**	P52732	326.27	52	54	Cytoplasm
RRBP1	Q9P2E9	296.76	31	33	Endoplasmic reticulum membrane
PRKDC	P78527	295.01	13	46	Nucleus
HSP7C	P11142	294.91	43	28	Cytoplasm, Nucleus
MYO1C	O00159	292.24	37	36	Cytoplasm, Nucleus
RBP2	P49792	288.28	15	39	Nucleus, Nucleus membrane
KI67	P46013	284.02	17	45	Nucleus
**FLNA**	P21333	278.61	18	38	Cytoplasm
BIP	P11021	273.17	46	27	Cytoplasm
HS90B	P08238	266.96	34	26	Cytoplasm, Nucleus
MYOF	Q9NZM1	266.44	16	29	Cell membrane , Nucleus membrane
UFO	P30530	264.07	28	19	Cell membrane
NOP56	O00567	263.73	50	28	Cytoplasm, Nucleus
K2C6B	P04259	255.8	51	34	Cytosol
HS90A	P07900	255.13	37	25	Cytoplasm, Nucleus
H2AY	O75367	248.19	55	16	Nucleus
TBB4B	P68371	241.16	51	17	Cytoplasm

Score[1]: Protein scores, calculated by Proteome Discoverer application from a list of peptides identified for a particular protein, indicate the relevance of a protein. Coverage[2]: Coverage of identified high-confidence peptides match the protein. Peptides[3]: Number of high-confidence peptides which match the protein.

Table S4: Top 20 candidates of KIF11-interacting proteins.

Protein name	Accession	Score[1]	Coverage[2]	Peptides[3]	Subcellular localization
MYH10	P35580	353.72	57	133	Cell projection
KIF11	P52732	299.26	50	63	Cytoplasm
LMNA	P02545	279.01	65	58	Nucleus
MAP1B	P46821	265.9	28	50	Cell projection, Cytoplasm
CKAP5	Q14008	247.81	30	52	Cytoplasm, Spindle
PARP1	P09874	225.87	30	29	Cytoplasm, Nucleus
K1C9	P35527	221.24	39	16	Cytosol, Nucleus
ACTH	P63267	216.18	32	17	Cytoplasm
SFPQ	P23246	213.18	28	23	Cytoplasm, Nucleus
H13	P16402	201.7	40	14	Nucleus
K1C18	P05783	195.53	46	20	Cytoplasm, Nucleus
COR1C	Q9ULV4	186.81	37	19	Cell membrane
LIMA1	Q9UHB6	180.9	27	17	Cell membrane
RS4X	P62701	180.64	37	16	Cytoplasm, Nucleus
NONO	Q15233	180.51	30	16	Nucleus
RFA1	P27694	176.71	30	14	Nucleus
SRPRA	P08240	171.16	20	11	Endoplasmic reticulum membrane
RECQ1	P46063	166.69	20	12	Nucleus
**CASR**	P41180	166.18	16	16	Cell membrane
RS7	P62081	157.96	56	10	Cytoplasm, Nucleus

Score[1]: Protein scores, calculated by Proteome Discoverer application from a list of peptides identified for a particular protein, indicate the relevance of a protein. Coverage[2]: Coverage of identified high-confidence peptides match the protein. Peptides[3]: Number of high-confidence peptides which match the protein.
